# Detection of bacteria using antimicrobial polymer derived via ring-opening metathesis (romp) pathway

**DOI:** 10.3906/kim-2012-14

**Published:** 2021-08-27

**Authors:** N. Ceren SÜER, Tülin ARASOĞLU, Hüsnü CANKURTARAN, Mustafa OKUTAN, Markus GALLEI, Tarik EREN

**Affiliations:** 1 Department of Chemistry, Faculty of Arts and Science, Yıldız Technical University, İstanbul Turkey; 2 Department of Molecular Biology and Genetics, Faculty of Arts and Science, Yıldız Technical University, İstanbul Turkey; 3 Department of Physics, Faculty of Arts and Science, Yıldız Technical University, İstanbul Turkey; 4 Chair in Polymer Chemistry, Saarland University, Saarbrücken Germany

**Keywords:** Sensors, bacteria, electrical properties, antimicrobial polymer, ROMP

## Abstract

There is growing interest in the detection of bacteria in consumables, for example, in the food and water sectors. In this study, the aim was to produce a polymer-based bacteria biosensor via ROMP (ring opening metathesis polymerization). In the first part of the study, block and random copolymers were synthesized, and their biocidal activities were tested on the glass surface. Interdigitated electrode arrays coated with the polymers possessing the highest activity were used to screen the affinity towards different bacterial strains by monitoring impedance variations in real-time. The polymer-coated electrode could detect gram-positive and gram-negative bacteria strains at a concentration of 10^7 ^cfu/mL. The results show that ROMP-based polymer offers bacterial detection and can be used in developing biosensor devices for efficiently detecting pathogenic bacteria.

## 1. Introduction

The evolution of antibiotic-resistant microorganisms is one of the biggest global threats [1–4]. Bacterial pathogens are the main cause of death in human and veterinary medicine worldwide. Research in recent years has shown that in developing countries, neonatal infections cause between 1.5 and 2 million deaths each year [5,6]. According to the Centers for Disease Control and Prevention, *Salmonella* causes sickness in 1.2 million Americans and leads to 23,000 hospitalization and 450 deaths every year. Around 1 million of these cases are caused by food sources *Salmonella* [online]. Website https://www.cdc.gov/salmonella/index.html. [accessed on 10 May 2019].1. Potable water is also another source for the spreading of infection. According to the WHO (World Health Organization), more than 5 million people die each year due to contamination from the food and water supplies by water-borne bacteria. These types of bacteria are known to cause diarrheal diseases *Salmonella Litigation Sources Water* [online]. Website https://salmonellalitigation.com/salmonella-sources/water-1. [accessed on 11 May 2019].2 [7]. *Staphylococcus aureus* ( *S. aureus* ) and *Escherichia coli* ( *E. coli* ) are particularly dangerous in hospital and medical settings [8]. Furthermore, *Salmonella spp* ., *Campylobacter spp.,* and *E. coli* are the primary/most widespread foodborne pathogens [4]. The presence of certain types of microorganisms can be costly and detrimental to human health, even resulting in death. Therefore, there is an increasing need for the development of new and simple methods for the detection of microorganisms in many different fields, such as in food production or water supplies.

Many techniques have been developed for bacterial detection. In recent years, biosensors have been playing an increasingly important role in the detection of pathogenic bacteria. The development of sensors is a high priority for real-time detection of bacteria that cause pollution [9,10]. Over the years, many biological procedures have been simplified, and faster methods have been developed for detection with significantly reduced test times and increased sensitivity. Most importantly, these advanced biotechnological analyses, called “rapid detection techniques”, have revealed new-generation analytical methods that are no longer based solely on the agar medium [11]. Current technologies for detecting microbial pathogens, such as enzyme-linked immunosorbent assay (ELISA), polymerase chain reaction (PCR), and pulsed field gel electrophoresis, offer quantitative, high-sensitivity measurements [5,12,13]. However, these methods are often time-consuming, requiring expensive laboratory facilities and equipment, as well as qualified technical experts [5]. Microelectromechanical system (MEMS) based biosensors have recently been developed for bacterial detection to avoid some of these problems [14]. In general, these technologies rely on antibodies for molecular recognition owing to their high specificity for antigen binding [15]. However, antibody-based MEMS biosensors have limited stability in high-temperature environments and require highly specific antibody-antigen pairs for each target [5]. For fast, inexpensive, high-precision analysis, various immunosensors, which are based on the antigenicity of the target bacterial cells, have been developed using fluorescence electrochemical impedance, quartz crystalline microbalance (QCM), and surface plasmon resonance (SPR) [5,16–20]. Pathogen biosensors developed to date mainly involve immunosensors and DNA-based detection. Biosensing based on aptamers (typically DNA- or RNA-based oligonucleotide sequences) is becoming popular for next-generation target receptors [21]. Nanomaterials can improve the performance of biosensors due to their unique physicochemical properties. In particular, nanomaterials can be effective in capturing analytes more efficiently during biosensing events due to their large surface area [22,23]. Especially, gold nanoparticles (AuNps) have been one of the nanomaterials widely used in the development of biosensors due to the possibility of surface functionalization and their outstanding optical and electrical properties [1].

Antimicrobial peptides (AMPs) have the advantages of good stability, simple synthesis procedures, and broad binding affinity towards microorganisms, making them particularly promising for pathogenic biodetection applications [17]. Although the exact mechanism through which they kill bacteria is not clear, AMPs have been reported to adhere to bacterial cells before breaking down the cell [24–26]. Based on this principle, several peptide-based biosensors have been developed in recent years [17]. Mannoor et al. reported improved bacteria detection using Magainin I based biosensors [19]. They used interconnected gold electrodes, and the binding activities between the peptide and the bacteria were monitored by electrochemical impedance spectroscopy (EIS). The results showed that Magainin I provided a detection limit of approximately 10^3^ cfu/mL for *E. coli* . Etayash et al. measured the impedance changes on covalently binding Leucocin A AMP to a gold electrode [11]. They produced reproducible impedance spectra that detected peptide-bacteria interactions at a concentration of 1 cell/μL. In addition, *Listeria monocytogenes* were selectively detected at a concentration of 10^3^ cfu/mL concentration in the presence of other Gram-positive strains. Jiang et al. immobilized Colicin V AMP to a gold electrode and used this as a bacterial sensor for samples of water contaminated with *E. coli* [27]. The sensor selectively detected *E. coli* at a concentration of 10^2^ cfu/mL. It has been proven that the sensor can detect *E. coli* in water samples at pH 7–9 and concentrations of 10^2^–10^6^ cfu/mL.

In recent years, synthetic mimics of host defense peptides derived from polymers have been extensively studied for their applications, especially in the fields of material science, biology, and biomedical science. Among them, cationic polymers, in particular, have several beneficial properties, such as a sustained inhibitory effect, broad-spectrum antibacterial activity, easy synthesis, and cost-effectiveness [28]. For example, a sensor surface coated with alternating by layers of oppositely charged polyelectrolytes (polyethyleneimine (PEI) and poly(sodium 4-styrenesulfonate) (PSS)) showed a detection limit as low as 10^1^ cfu/mL for *E.coli* with a response time of around 20 min [29]. Previously we observed that a pyridinium and phosphonium-bearing ROMP type polymer showed biocidal activity on a solid surface [30,31]. We think an electrode surface covered with a biocidal ROMP-type polymer could be used as a bacteria sensor. Cationic polymers are preferentially more robust than peptides in environmental conditions [32], so coating a gold electrode with cationic biocidal polymers can provide increased shelf life and lower costs compared to antimicrobial peptide-coated electrodes [33–35]. 

Here, biocidal active triphenylphosphonium-thioacetate or hexylpyridinium-thioacetate polymer series were coated on gold electrode to be used as a bacteria sensor. The interaction between the bacterial surface and the antimicrobial polymer was measured using electrochemical impedance spectroscopy and cyclic voltammetry.

## 2. Materials and methods

### 2.1. Materials

Furan, maleic anhydride, 3-bromopropylamine hydrobromide, 1-bromohexane, 3-aminomethyl pyridine, triphenylphosphine, cysteamine, hydrogen tetrachloroaurate(III), sodium borohydride, ethylvinyl ether, 3-bromopyridine, acetic anhydride, petroleum ether, diethyl ether, *N,N* -dimethylformamide (DMF), *N,N* -dimethylacetamide (DMAc), dichloromethane (DCM), chloroform, pentane, dimethyl sulfoxide (DMSO) were purchased from Sigma Aldrich and used as received. Ethyl acetate and hexane were distilled by conventional techniques. Grubbs second generation catalyst was purchased from Sigma Aldrich. Grubbs third generation catalyst [(H_2_-Imes)(3-Br-py)_2_-(Cl)_2_Ru=CHPh] was freshly prepared according to the previously reported procedure [38]. Phosphate buffered saline (PBS) was obtained from Sigma Aldrich in tablet form. Mueller–Hinton broth (MHB) and Mueller–Hinton agar (MHA) media (both from Dıfco, BD Diagnostic Systems, United States) were used for microbiological tests. *Staphylococcus aureus* ATCC 25923 strain and *Escherichia coli* ATCC 25922 strain were acquired from the department of Molecular Biology and Genetics Department (Yildiz Technical University, Turkey) and used as representative of Gram-positive and Gram-negative bacteria, respectively (ATCC is the registered trademark of American Type Culture Collection).

### 2.2. Instrumentation

^1^H NMR (500 MHz), ^13^C NMR (75 MHz), and ^31^P NMR spectra were recorded using a Bruker Avance III 500 MHz spectrometer. The appropriate frequencies using either residual CDCl_3_ or DMSO *-d* *_6_* as internal reference (for ^1^H and ^13^C) were applied for the analysis of NMR data. Perkin–Elmer Spectrum One spectrometer was used for attenuated total reﬂectance Fourier transform infrared (ATR–FTIR) spectroscopy. Impedance Spectroscopy was measured by using Hioki 3532 LCR meter at room temperature. Size-exclusion chromatography (SEC) was performed with a system consisting of a Waters 515 pump, a RI Waters 2410 and PSS GRAM 1000 column. DMF (with LiBr, 3 g L^–1^) was used as the mobile phase (flow rate 1 mL min^–1^) at 60 °C. Pullulans from PSS (Polymer Standard Service (PSS), Mainz, Germany) were used as standards. SEM analysis was performed using a Philips XL30 ESEM-FEG/EDAX instrument by the backscattered electrons (BSE) and the secondary electrons (SE) detectors. The measurements of TEM were carried out on a JEOL JEM-2100 with a LaB6-cathode (200 kV). Camera is a Gatan Orius SC1000 CCD. CV measurements were carried out using a Radiometer Analytical PST050 potentiostat, interfaced to PC running Voltamaster 4.0 software. Absorbance spectrum was recorded using a Shimadzu UV-2600 Spectrometer.

### 2.3. Synthesis 

#### 2.3.1. Synthesis of compound 1

The compound 1 was synthesized according to literature [37]. 63 g (642 mmol) of maleic anhydride was dissolved in THF and 48 mL (660 mmol) of furan was added by pipette to the reaction medium. After mixing under nitrogen gas for 5 min, the reaction was stopped and kept at room temperature in the dark for 4 days. White oxanorbornene crystals were seen in the reaction vessel after 4 days. For purification, crystals were washed with cold THF. Diels-Alder product all obtained as exo-oxanorbornene (yield: 78%).

#### 2.3.2. Synthesis of compound 2

The compound 2 was adapted from literature [38]. NaHCO_3_ (5.44 g, 65 mmol) was dissolved in 50 mL of water in the reaction vessel. 3-bromopropylamine hydrobromide (14.24 g, 65 mmol) was added slowly onto the solution. After stirring for a few minutes, compound 1 (10 g, 60.24 mmol) was added to the reaction medium, and the reaction was stirred at room temperature for 30 min. It was purified under vacuum washing with water and diethyl ether and dried in a vacuum oven. In the second step, the suspension of sodium acetate (0.72 g, 8.28 mmol) and acetic anhydride (16 mL) was stirred in a 2-necked flask at 90 °C for 10 min. The white product (4 g, 13.16 mmol) synthesized in the first step was added onto this mixture and stirred at 90 °C for 1 h. After 1 h, the solution in the reaction vessel was poured into 20 g of ice in the beaker and 50 mL of chloroform was added. The chloroform phase was with 3 × 30 mL of 5% NaHCO_3_ and with 30 mL of 10% NaCl, then dried by adding MgSO_4_, filtered and the solvent was removed by evaporator. It was purified by column chromatography (ethylacetate: hexane 1:1 v/v) and a white solid was obtained (yield: 40%). 

#### 2.3.3. Synthesis of monomer 1

The monomer 1 was synthesized according to literature [39]. Compound 2 (0.5 g, 1.75 mmol) and triphenylphosphine (1.38 g, 5.24 mmol) were mixed in a sealed tube with 11 mL of ethyl acetate at 50 °C under nitrogen gas for 24 h. After 24 h, the reaction vessel was cooled to room temperature; product was purified by washing with ethyl acetate, THF, and diethyl ether and dried under nitrogen (yield: 30%).

#### 2.3.4. Synthesis of monomer 2

The monomer 2 was synthesized according to literature [40]. In the first step, 2.4 mL (22 mmol) of 3-aminomethylpyridine, 1.92 g (11 mmol) of compound 1, and 6 mL of DMAc were placed in a flask and stirred at 60 °C for 20 min. A catalytic amount of sodium acetate (0.5 g, 5 mmol) was dissolved in 12 mL of acetic anhydride and added to the mixture. The reaction temperature was raised to 90 °C and stirred for a further 2 h. After the mixture was cooled to room temperature, it was diluted with ethyl acetate, followed by extraction with 10% NaCl solution. To purify the product, a white solid was obtained by column chromatography with the aid of THF: hexane (3:1, v/v) solvent (yield: 50%). After white solid (0.52 g, 2 mmol) was placed in a single neck reaction flask, 12 mL acetonitrile was added to it and dissolved at room temperature. 1-bromohexane (1.57 g, 9.51 mmol) was added onto this solution and stirred under nitrogen gas and reflux at 60 °C for 24 h. The resulting product was precipitated in 200 mL diethylether. It was washed with diethyl ether by centrifugation and removed from the solvent under vacuum and dried under nitrogen (yield: 60%). 

#### 2.3.5. Synthesis of monomer 3

Compound 1 (2.0 g, 12 mmol) was dissolved in 6 mL dry DMAc. Cysteamine (1.11 g, 14.4 mmol) was dissolved in 10 mL dry DMAc and added to the mixture with syringe under nitrogen at room temperature and stirred for 3 h. Mixture of a catalytic amount of sodium acetate (0.44 g, 5.44 mmol) and 13 mL acetic anhydride was added to the reaction medium and stirred for overnight at 55 °C. After cooling the reaction mixture to room temperature, the solution was diluted with ethyl acetate, extracted using brine. The product was purified by column chromatography using ethyl acetate:hexane (1:1, v/v) (yield: 35%).

^1^H NMR (500 MHz, CDCl_3_) δ 6.49, 5.24, 3.68, 3.67, 3.66, 3.10, 3.08, 3.07, 2.82, 2.29.

^13^C NMR (126 MHz, CDCl_3_) δ 194.72, 175.65, 136.39, 80.69, 47.30, 37.85, 30.35, 26.43.

### 2.4. General polymerization procedure

In a typical example, freshly prepared Grubbs third generation catalyst was dissolved in 0.5 mL of dry dichloromethane (DCM) and added in the vigorously stirring monomer 1 which was dissolved in 2 mL dry chloroform. After the reaction mixture was stirred for 2 h at room temperature, monomer 3 in 0.5 mL of dry chloroform was added to the reaction medium. The reaction was then terminated by an injection of 0.5 mL 30% ethylvinyl ether (in DCM). The copolymers were precipitated and washed with diethyl ether and dried under nitrogen. All the copolymers were dissolved in DMSO *-d* *_6_* for NMR characterization. For the copolymerization of monomer 2 and monomer 3; monomer 2 was dissolved in dry DMF. Firstly, monomer 2 was stirred overnight and confirmed its disappearance by TLC then monomer 3 was added to the solution to obtain the theoretical molecular weight of 10,000 g/ mol. 

### 2.5. Determination of the solid phase antibacterial activity

#### 2.5.1. Glass surface coating

Spin coating techniques are used for the glass surface coating with the polymers for further antibacterial assay. The glass surfaces were cleaned using Piranha solution (30% H_2_O_2_/70% H_2_SO_4_) for 5 min. Afterwards, the slides were thoroughly rinsed with firstly distilled water and later isopropanol and then dried under an air atmosphere [41]. Solutions of copolymers in DMSO (8 mg/mL) were coated on the glass slides using a spin coating technique at 2000 rpm for 30 s. The copolymer-coated glasses were dried under vacuum for 2 h to evaporate any remaining solvent at 70 °C. Samples were then applied for antibacterial analysis. 

#### 2.5.2. Preparation of bacterial suspensions 

The bacterial suspensions used in the study were prepared by suspending a few well-isolated colonies from MHA plates into MHB and incubated at 37 °C and 200 rpm in a shaking incubator for an overnight period. The suspensions were then diluted with the sterile buffer solution until it has an absorbance of 0.28 ± 0.02 at 475 nm, as measured spectrophotometrically. This measured value corresponds to the concentration of 1.5–3.0 × 10^8 ^cfu/mL that is used as the working dilution of bacterial inoculum in the antimicrobial methods applied in this study.

#### 2.5.3. Antibacterial activity test 

The antimicrobial sustainability of samples coating on microscope slides was performed according to the literature [42]. Briefly, 10 µL volume of the working dilution of each test bacteria prepared as mentioned above was dropped onto the surface of 25 mm diameter antimicrobial polymer coated glass materials. The inoculated glass materials were incubated at 37 °C and over 90% relative humidity for 1 h. Then, in order to recover the microorganisms that remained alive on the surface of the glass materials, they were transferred to sterile containers with 10 mL of MHB medium. This container was kept at room temperature for 30 min. At the end of the period, 10-fold serial dilutions were made by taking samples from these containers. Samples of 100 µL were taken from each dilution and spread onto MHA agar plates with swab. The plates were incubated for 24 h at 37 °C and each of the bacterial colony were counted and expressed as colony forming units per mL (cfu/mL). In this study, 25 mm diameter empty glass material and DMSO coating glass material were used as control samples. The results are determined as; 

log reduction = log (cell count of control) – log (survivor count on test samples glass materials).

### 2.6. Impedance spectroscopy analysis

#### 2.6.1. Electrode coating

The electrode surfaces were cleaned using sonication in isopropanol for 30 min and later dried under argon gas. Ten µL solutions of copolymers in DMSO (8 mg/mL) for impedance and cyclic voltammetry analysis were dripped on electrode surface and dried for 15 h to evaporate the solvent at 70 °C.

#### 2.6.2. Impedance measurements

The complex impedance is usually defined as Z* (ω) = Zʹ (ω)-iZʹʹ (ω) = R(ω) - iX(ω) (1). Here, ω = 2π *f* is angular frequency, *f* is frequency, Zʹ(ω) is real part, and Zʹʹ(ω) is imaginary part of the complex impedance. The complex impedance Z as a function of angular frequency is illustrated below for the absolute impedance; . Frequency dependent impedance values measurements made in the room temperature RMS 0.1 voltages with interdigital transducer (IDT) sensors.

After UV absorbance measurement, freshly prepared bacterial solutions were used for analysis immediately.

### 2.7. Cyclic voltammetry

3b coated electrodes were put into the PBS and 0.01 M K_4_[Fe(CN)_6_] (prepared in PBS) solutions, respectively. Later cyclic voltammograms were measured. Same electrodes were kept in *S. aureus* and *E. coli* solutions at the concentration of 10^7^ and 10^5 ^cfu/mL for 15 min. Afterwards, the electrodes were placed in the K_4_[Fe(CN)_6_] solution and the cyclic voltammograms were recorded. A new 3b coated electrode was used for each measurement. The measurements were recorded at a scanning speed of 100 mV s^–1^ and in the range of –400–(+800) mV. In the cyclic voltammetry metage, IDE (interdigitated electrode), platinum wire, and calomel (3 M KCl) electrode were used as working, auxiliary and reference electrodes, respectively.

## 3. Results and discussion

**Scheme 1:**
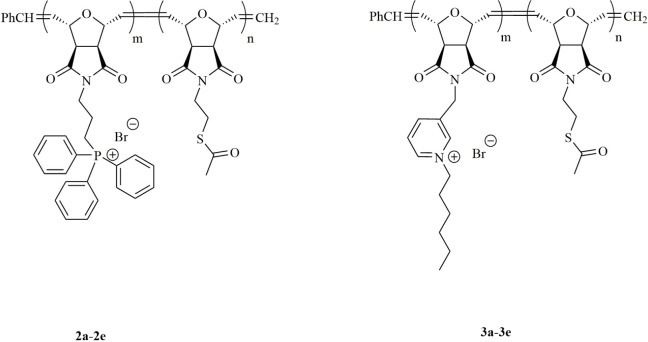
Polymers used in this study.

In this work, we first evaluated the biocidal activity on a glass surface of copolymers with triphenylphosphonium-thioacetate (polymer series 2a–e) or hexylpyridinium-thioacetate (polymer series 3a–e) functionalities. The presence of triphenylphosphonium or hexypyridinium moieties enhances biocidal activities of the copolymer, while thioacetate group favors the binding on the electrode surface. Scheme 1 shows the polymers synthesized in this study. 

### 3.1. Monomer synthesis

**Scheme 2:**
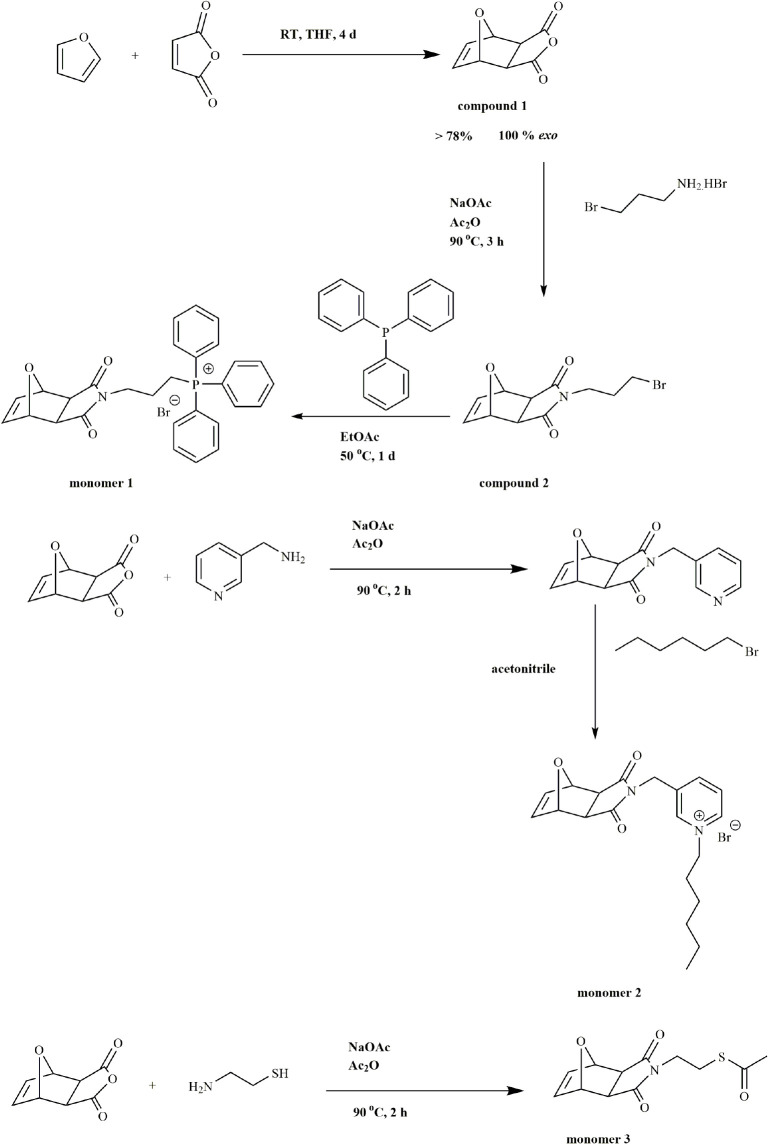
Synthesis of monomer 1, monomer 2, and monomer 3.

Monomer 1 and monomer 2 were synthesized and characterized according to methods described in the literature as shown in Scheme 2 (Figure S1 and S2) [37–39,43]. Synthesis of monomer 3 was described in SI and analyzed using NMR spectroscopy (Figure S3 and S4). Imide formation and acetylation of thiol group were conducted in the presence of catalytic amounts of sodium acetate [46]. Characteristic signals of oxanorbornene protons were observed at δ 6.49, 5.24, and 2.82 ppm and the protons of the substituent neighbor to the sulphur appeared at δ 3.08 in the ^1^H NMR spectra (Figure S3). The protons of the acetyl group at the end of the monomer were observed at δ 2.29 ppm. ^13^C-NMR spectrum of the monomer 3 showed thioacetate carbonyl (-S-C(=O)-CH_3_) at δ 194.7 ppm (Figure S4). Imide carbonyl of oxanorbornene was observed at 175.6 ppm. Olefinic carbons of oxanorbornene were detected at 136.4 ppm. Methyl carbon of thioacetamide group showed a peak at δ 26.43.

### 3.2. Polymer synthesis

Previously, a triphenyl phosphonium-possessing homopolymer derived via ROMP was shown to have antibacterial properties in solution as well as on a solid surface [31,39]. Hexylpyridinium bearing polymers were also proved to feature antibacterial properties on a glass surface [31,43]. From these previous studies we have obtained, if we can make copolymer series containing these functional groups, we can obtain a biocidal effective copolymer series similarly. For this purpose, copolymer series containing thioacetate functional group was synthesized. Thioacetate group is thought to be helpful in gold nanoparticle synthesis [45] and enhances the bonding of copolymers to the gold electrode surface [46].

**Scheme 3:**
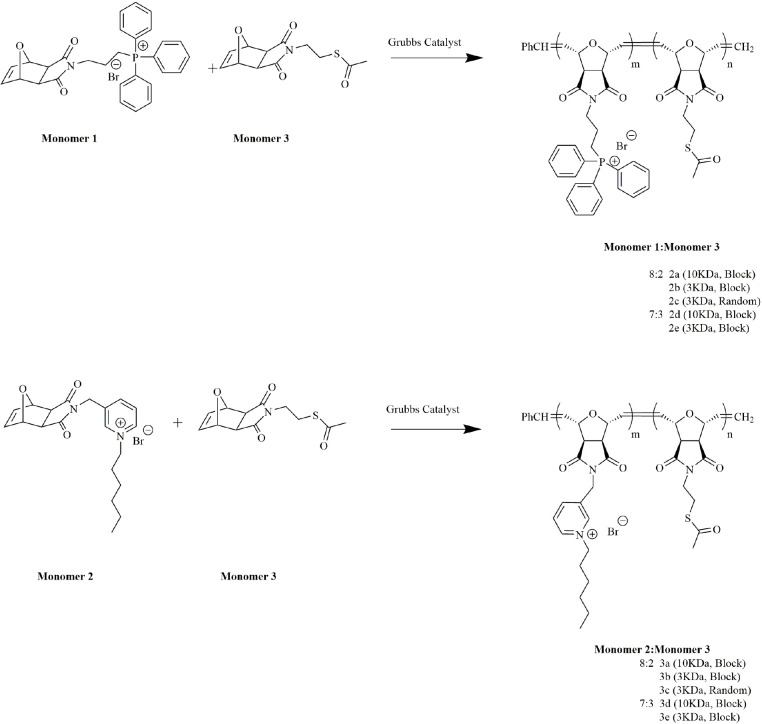
Synthesis of copolymers series 2a-2e and 3a-3e.

Thus, the random and block copolymers of triphenylphosphonium or hexylpyridinium monomer and thioacetate units with a target molecular weight of 3000 or 10,000 g/mol were synthesized via ROMP using the Grubbs 3rd generation catalyst. The monomers and their compositions used in polymer series are shown in Scheme 3. The cationic functionality and thioacetate residue were obtained via in the copolymers at ratios of either 8:2 or 7:3 by weight. Copolymer series 2a–2e possesses triphenylphosphonium and thioacetate functional groups, while copolymer series 3a–3e contains hexylpyridinium and thioacetate functional groups. Block copolymers were achieved by sequential addition of each functional monomer. After adding the catalyst to a vial containing a solution of monomer 1 or monomer 2, the polymerization of the first block went to completion, as confirmed by thin layer chromatography (TLC). Subsequently, a solution of monomer 3 was added to the polymerization medium in order to obtain the thioacetate functionality after cleaving at the end of the copolymerization step. 

^1^H NMR spectra of copolymers are shown in the supporting information Figures S5–S17. Specifically, the peak at 6.49 ppm for the double bond (-CH=CH-) of the oxanorbornene ring disappeared as a result of polymerization and turned to a/b *trans/cis* at between 6.00 and 5.50 ppm as shown in Figure 1 and Figure 2 [39]. 

**Figure 1:**
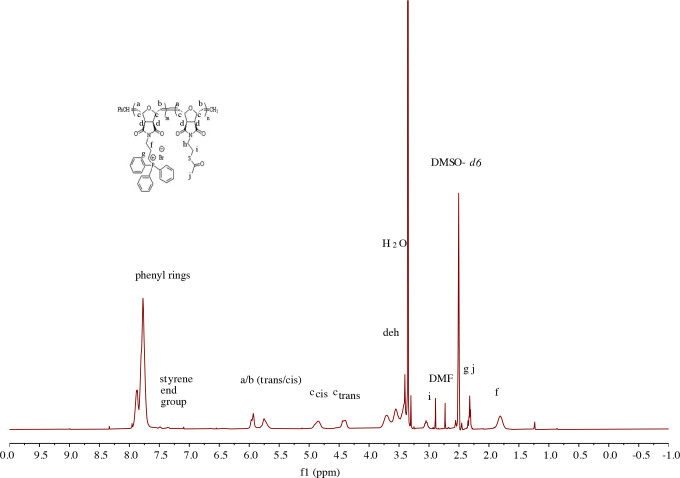
^1^H NMR spectrum of block copolymer 2a (M_n,th_ = 10,000 g/mol with a theoretical ratio m:n (8:2) by weight) in DMSO-d_6_.

**Figure 2:**
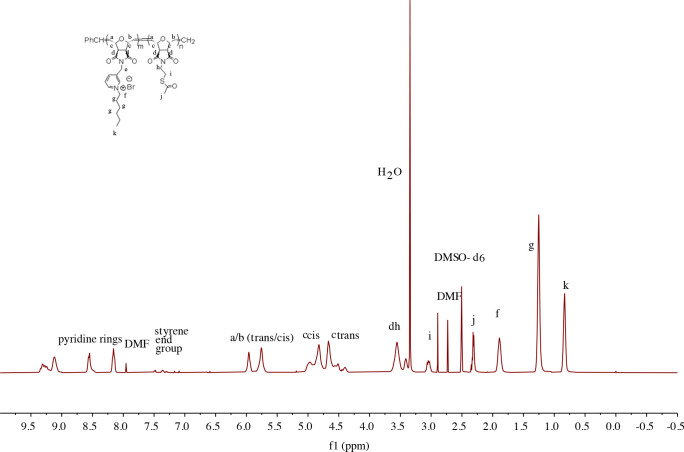
^1^H NMR spectrum of block copolymer 3a (M_n,th_ = 10,000 g/mol; with a theoretical ratio m:n (8:2) by weight) in DMSO-d_6_.

Table 1 shows the observed feed ratios and molecular weight of the copolymer series. The ratio of the number of repeating units of the monomer 1 or 2 block (m) to the number of repeating units of the thioacetate block (monomer 3) (n) was analyzed by comparing the corresponding ^1^H NMR peaks. As a typical example, the m and n values of copolymer 2b are calculated here, and the same procedure was applied for all series of copolymers. The methylene group (-CH_2_-) of the phenyl phosphonium block appears at 1.81 ppm and contain 2 proton units. The styrenic end group stemming from the Grubbs 3rd generation catalyst appears between 7.5 and 7.2 ppm and contains 5 proton units. The ratio of the integrations of these peaks resulted in an m value of 3.5. The alkyl group (-S-CH_2_-) appears at 3.05 ppm and contains 2 proton units. The ratio of the alkyl group and the styrenic end group integrals gives an n value of 2.9. 

**Table:** Table. Characterization summary of the copolymers.

Polymer Code	Polymer	ma	nb	m/nc	m/nd	Mne	Mnf	Mng	Ð h
						(g/mol)	(g/mol)	(g/mol)	
(2a)	monomer 1: monomer 3 (block)	37.6	13.1	1.9	2.8	10,000	24,122	4887	1.18
(2b)	monomer 1: monomer 3 (block)	3.5	2.9	1.9	1.2	3000	2694	2187	1.21
(2c)	monomer 1: monomer 3 (random)	3.9	3.3	1.9	1.2	3000	3020	2381	1.17
(2d)	monomer 1: monomer 3 (block)	27.6	20.1	1.1	1.4	10,000	20,528	4346	1.18
(2e)	monomer 1: monomer 3 (block)	3.9	4.3	1.1	0.9	3000	3288	2248	1.3
(3a)	monomer 2: monomer 3 (block)	19.3	7.5	1.9	2.5	10,000	10,136	4620	1.31
(3b)	monomer 2: monomer 3 (block)	5.45	2.7	1.9	2.0	3000	3017	2300	1.15
(3c)	monomer 2: monomer 3 (random)	8.5	2.9	1.9	2.9	3000	4356	2812	1.25
(3d)	monomer 2: monomer 3 (block)	23.6	12.5	1.1	1.8	10,000	1284	4113	1.45
(3e)	monomer 2: monomer 3 (block)	7.3	4.6	1.1	1.5	3000	4305	2186	1.21

a,b Calculated degree of polymerization (DP) of monomers by 1H NMR.cTheoretical mole ratio of monomers in the copolymer.dCalculated mole ratio by 1H NMR.eTheoretical number-average molecular weight.fCalculated number-average molecular weight by 1H NMR.gDeterminated number-average molecular weight by SEC. hDeterminated polydispersity index by SEC in DMF + salt vs. pullulan standards.

The values of m and n give the degree of polymerization for each block in a chain of polymer. By multiplying these m and n values by molecular weight of the monomers, the average molecular weight of the polymer is obtained. For example, targeted molecular weight of 2b was 3000 g/mol and the observed molecular weight (3.5 × molecular weight of monomer 1 (548.42 g/mol) + (2.9 × molecular weight of monomer 3 (267.3 g/mol) = 2694 g/mol). 

For polymer series 3a and 3b, it was observed that thioacetate-based monomer did not bind to the polymer chain in high efficiency. There could be several reasons for this observation. One could be that polymerization was conducted under DMF and DMF strongly binds to the catalyst center that retard or inhibit polymerization efficiency. Second plausible explanation is that hexylpyridinium possessing growing chain form a micellar formation in the polymerization conditions that might retard the second monomer incorporation to the ruthenium center. This was especially observed in the synthesis of high molecular weight (targeting molecular weight of 10,000 g/mol) block copolymer series. When the concentration of monomer 3 is increased (feed ratio increasing to 7: 3), it has been observed that monomer 3 is added to the polymer chain, even if slightly. Appearance of ^1^H NMR signal at 3.0 ppm related to the protons of (-C *H*_2_-S(=O)-). Another important result seen in Table 1 was significant deviation in molecular weights according to end group analysis in 2a and 2d block copolymer series. It is thought that the reason for this is that cross metathesis results in formation of high molecular weight polymer.

Size-exclusion chromatography (SEC) analysis was performed for all copolymers. Polymers were measured via SEC using DMF as the mobile phase vs. pullulan standards. However, the average molecular weight ( *M*_n_) calculated by NMR end group analysis of the copolymers did not agree with the *M*_n_ values determined by SEC (Table 1). The *M*_n_ results determined by SEC were lower than the *M*_n_ values obtained by NMR end group analysis. The linear, nonionic pullulan standards used for SEC calibration may have resulted in different hydrodynamic volumes compared to cationic-based copolymers, which can lead to a strong deviation [49]. Nevertheless, predominantly monomodal distributions were observed with *Ð* between 1.15 and 1.45. GPC graphs of polymers were given in Figure S18 and Figure S19. GPC chromatograms showed a distinct shoulder for the series of block copolymer 2e, 3d, and 3e. This could be due to the uncontrolled block copolymer formation possible intermolecular or intramolecular cross metathesis occurring during the addition of the second block and low initiation of macro initiators. 

After the characterization of polymer, antibacterial activity of these series was conducted to investigate the structure-property relationship. The effect of phosphonium and pyridinium salts possessing different hydrophobicities as well as thioacetate moieties might also induce difference in biocidal activity on the surface. The following section gives brief discussion on antibacterial activity of the synthesized polymers on glass surface.

### 3.3. Antibacterial activity

In polymer-based sensor development study, first of all, antibacterial properties of polymers on the solid surface were examined. After finding the most effective polymers on solid surface, electrode surfaces were coated with these polymers and then the sensor feature was studied. Therefore, antibacterial activity of 2a–e and 3a–e was tested on a glass surface. Before the test, surface of 2.5 × 2.5 cm glass microscope slides was cleaned using Piranha solution. Polymer solutions were poured onto separated slides and coated by spin coating at 2000 rpm for 30 s. The slides were dried under vacuum at 70 °C for 2 h to evaporate any remaining solvent. The surfaces were then tested for efficacy at killing two significant pathogens: Gram-negative bacteria *E. coli* and Gram-positive bacterium *S. aureus* . The antimicrobial sustainability of samples coating on microscope slides was performed according to the literature.[44] The polymer coated glass surface were challenged with *E.coli* and *S. aureus* at a concentration of about and the plates were incubated for 24 h at 37 °C and counted for colony-forming units. In the study, 25 mm diameter empty glass material (NC) and solvent (DMSO) coated glass material (SC) used as control samples. The empty glass materials containing only bacteria were used as a cell count of control in the log reduction and % killing calculations. Three polymer coated glasses were analyzed for each bacteria species. Average results were obtained with 3 replicates of each analysis.

**Figure 3:**
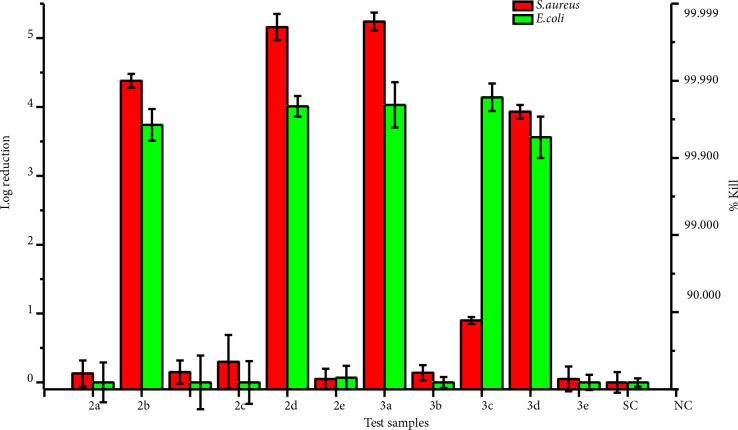
The log reduction and kill% results for S. aureus and E. coli on the test samples

Figure 3 shows log reduction and kill% results for *S. aureus* and *E. coli* on polymer-coated glass surface for the polymers that were used to cover the gold electrode later. Figure 3 indicated that the 3a–e series was more potent than the 2a–e series (see the Petri images of surviving bacteria in Figure S20). Block copolymer series 3e and 3b with 2e and 2b possessing 3000 g/mol molecular weight showed higher activities against *E.coli* and *S. aureus* than the other copolymers. There could be several reasons for these results. Copolymers gaining lower activity tend to stick to each other because of the strong hydrophobic and π-π interaction of the phenyl rings and presumably form polymer aggregates on the surface. Thus, such aggregates mean that the polymer is unable to interact with the bacterial membrane efficiently to overcome electrostatic interaction with bacterial membrane. 

As seen in Figure 3, the highest antibacterial activity was detected against both organisms for copolymer 3b with a 5.24 log reduction and 99.999% kill activity for *S. aureus* and a 4.03 log reduction and 99.989% kill activity for *E. coli* . While log reduction was 4.38 and killing activity 99.996 % for copolymer 2b against *S. aureus* , log reduction and killing activity were 3.74 and 99.98% for *E.coli* , respectively. Interestingly, while the antibacterial activity of polymer 3d against *S. aureus* was low (0.9 log reduction), it was quite high against *E. coli* (4.14 log reduction). The second highest antibacterial activity was detected for copolymer 2e with a 99.999% killing activity 5.16 log reduction for *S. aureus* and a 4.01 log reduction 99.99% kill activity for *E. coli* . 

Low molecular weight polymers, 2e, 2b, 3e, and 3b, have a higher degree of freedom inside the polymer matrix and fewer entanglements. Thus, it is supposed to feature a higher entropy under physiological conditions. More freedom on the surface allows for easier penetration or interaction with the bacteria membrane than a higher molecular weight polymer, which is thermodynamically more difficult to move on the surface. The biocidal activity and relationship with the degrees of freedom on the substrate surface were also reported by Lienkamp and co-workers [50]. 

It was observed that the polymer-coated surfaces showed higher activity against *S. aureus* than *E.coli* , except the copolymer 3d, which was in agreement with results reported in the literature [33]. This situation is attributed to the different cell wall structures of *E. coli* and *S. aureus* .

### 3.4. Gold nanoparticle synthesis

We aimed that the incorporation of gold cluster into the polymer matrix could enhance impedance signal. Thus, we performed the biocidal activity of gold nanoparticles incorporated in the copolymer series 2b, 2e, 3b, and 3e.

Cationic phosphonium and ammonium gold nanoparticles were synthesized according to literature [47]. In a typical example, polymer 2b (0.2 g, 0.06 mmol) was dissolved in DMSO (4.24 mL x 2.24) (2.24 = theoretical DP of thioacetate group) and stirred vigorously at room temperature. A solution of hydrogen tetrachloroaurate(III) (0.02 mmol x 2.24 (theoretical DP of thioacetate group DP)) in DMSO (2.12 mL x 2.24 (theoretical DP of thioacetate group DP)) was added to polymer mixture. The reduction was accomplished by adding freshly prepared aqueous (1.06 mL x 2.24 (theoretical DP of thioacetate group)) sodium borohydride (2.0 mmol) solution to the solution of reaction. Dialysis membrane (100–500 Da) was applied to purify the product. After that, gold nanoparticle bearing polymer 2b* was dried in oven overnight at 60 °C. 

 It was observed that introducing gold nanoparticles in the copolymer series resulted in complete loss of biocidal activity of these polymers on the glass surface against both *E.coli* and *S. aureus* . After the synthesis of gold nanoparticles, it is predicted that the polymers cover the gold surface, and the overall cationic charge and hydrophobic tail groups change. This causes an alteration of polymer morphology on the surface, and as a result, the biocidal activity is lost.

### 3.5. Polymer-coated electrode surface

As polymer 3b showed the highest activity on the glass surface against *S. aureus* and *E. coli* (5.24 and 4.03 log reduction, respectively), it was further used to coat the electrode surface. Here, electrode surface was also coated with one of the active copolymer series, 2b, which is possessing phosphonium functionality and having the same cationic units and molecular weight with respect to hexylpyridinium polymer 3b to investigate the structure-property relationship for each series of copolymer. Electrode surface coating with polymer was performed by spin coating and drop-cast coating techniques. However, it has been observed that the drop cast technique is a better method for covering the surface. The adsorption of cationic polyelectrolytes possessing thioacetate group on gold electrode surface is type of method for surface modification. Driven force for adsorption on the Au surface is entropy via macroions and could also be constituted directly from thioacetate group without using a base [46].

**Figure 4:**
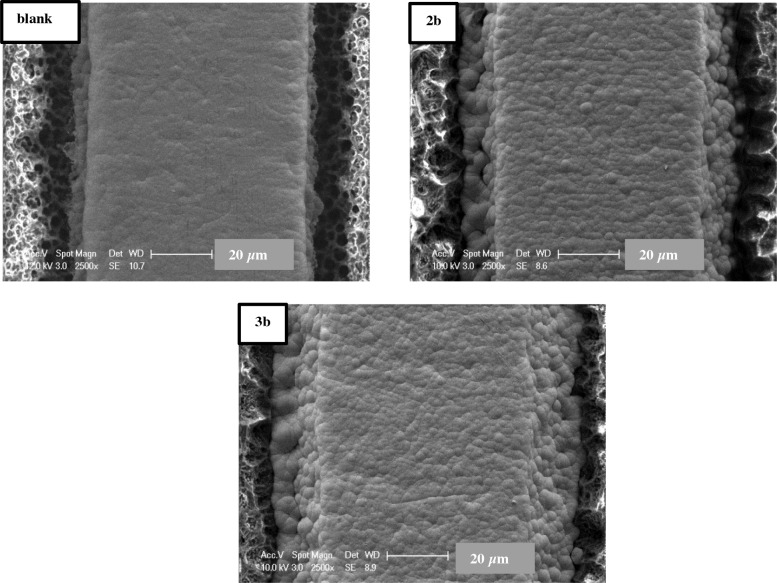
SEM images of uncoated, 2b, and 3b coated electrode surface, respectively.

We confirmed the coating of the electrode surface by FTIR and scattering electron microscope (SEM) analysis. The FTIR spectrum (Figure S21) showed the appearance of thioacetate and amide stretching at 1714 and 1705 cm^–1^, respectively. Figure 4 shows SEM images of the uncoated control electrode (blank), electrode coated with 2b and 3b, respectively. When we investigated the SEM image of 2b or 3b gold electrode, spheroidal layer on the electrode surface indicated to us the surface of the gold electrode was covered with 2b or 3b while uncoated gold electrode surface was smooth.

### 3.6. Electrochemical impedance spectroscopic measurements

The sensor element is an impedimetric transducer with two planar interdigitated electrodes that is known as an interdigitated electrode array (IDEA) [49,50]. IDEA consists of a series of parallel planar electrodes with alternating electrodes connected together, forming an interdigitated electrode. Adhesion of the bacteria on the polymer-coated electrode surface affects the interfacial electron transfer process and decreases the electron transfer on the surface, resulting in increased capacitance depending on the number of bacteria [51]. Negatively charged bacteria species can adhere to the cationic polymer-coated electrode surface, causing variations in the electrical impedance. Attachment of the bacterial cell and disruption of the bacterial membrane in time scale minutes to tens of minutes may affect the charge resistance and conductivity in faradic impedance measurements. 

The gold electrodes coated with biocidal cationic polymers (2b and 3b) were applied for impedance measurements in phosphate-buffered saline solution (PBS) and bacteria solution. The objective was to explore the feasibility of employing antimicrobial polymers in impedance analysis for sensitive detection of *E. coli* and *S. aureus.*

The sensitivity and selectivity of bacterial detection is an important parameter for the practical usage of the sensor. We analyzed the impedance frequency range from 10 Hz to 1 kHz (see supporting information Figures S22-S23 and S24-S25 for the polymer series 2b and 3b, respectively) for the electrode sample coated with 2b and 3b **,** respectively. Thus, the sensitivity of the 2b and 3b polymer-coated sensors were investigated in the presence of different concentrations of bacteria (10^3^, 10^5^, and 10^7^ cfu/mL). In each experiment, a blank sample (i.e., buffer media with no bacteria) was also subjected to impedance readings for comparisons. Furthermore, frequency response shows maximal changes at a lower frequency (100 Hz); we conducted that the time-response graph of electrode impedance in response to bacteria at this frequency. The real-time impedimetric response of the copolymer 2b and 3b coated sensor to various concentrations of *S. aureus* and *E. coli* was investigated at spot frequency 100 Hz and reported in Figure S26 and Figure S27. 

Figure S26 shows the results of measurements performed after incubation of the polymer sensor with *S. aureus* and *E. coli* for copolymer 2b. The results show that the impedance signal increased logarithmically with increasing *S. aureus* concentration compared to the blank. The impedance response is proportional to the serial diluted bacterial cell, and the signal was in the order 10^7^ > 10^5^ >10^3^ cfu/mL. The limit of detection of this polymer-coated electrode device for *S. aureus* was found to be 10^7^ cfu/mL, which is below the clinically significant for controlling bacterial growth (10^3^ cfu/mL) [52]. 

Likewise, when analyzing *E. coli* samples, signal reported in Figure S27 was in the same order 10^7^ > 10^5^ > 10^3^ cfu/mL and follow the concentration. *E. coli* at a concentration of 10^3^ cfu/mLexhibits the impedance value was similar to that of the blank sample, i.e., buffer with no bacteria. We thought that the binding kinetics on the electrode surface is crucial for obtaining the proper signal. The dielectric characteristics obtained for the outer membrane of *E. coli* show that it has a very low conductivity of 2 × 10^–6^ S m^–1^ [53]. Since the binding of *E. coli* to the electrode surface is slower, the free *E. coli* cells slightly increase the conductivity of the solution, resulting in a slightly decline signal to the blank (buffer, no bacteria). In contrast, we observed a response to *S. aureus* at the same concentration. This could be attributed to the better biofouling and adhesion properties of *S. aureus* on the electrode surface over time. 

**Figure 5:**
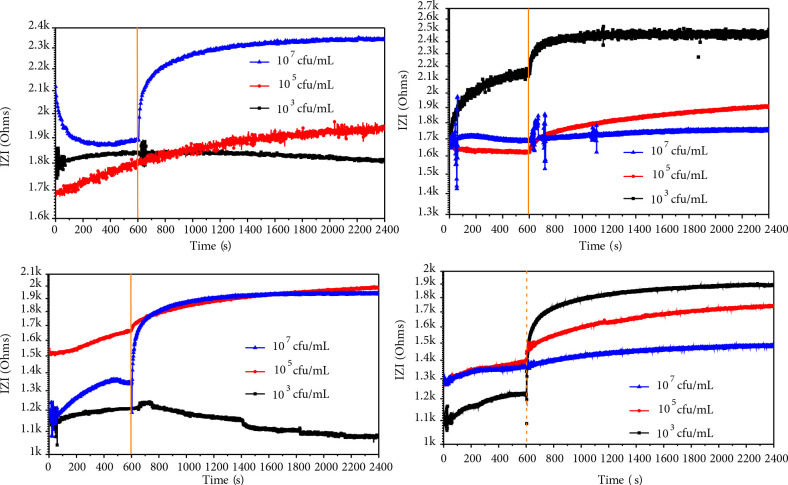
(a) Impedance-time graph of the polymer 2b based sensor against E. coli; (b) Impedance-time graph of the polymer 2b based sensor against S. aureus; (c) Impedance-time graph of the polymer 3b based sensor against E. coli; (d) Impedance-time graph of the polymer 3b based sensor against S. aureus with different concentrations

Figure 5 shows the impedance-time graph of the polymer 2b and 3b based sensor against *E. coli* and *S. aureus* . Although all the investigated frequencies exhibited clear impedimetric responses to *S. aureus* and *E. coli* cells, 100 Hz appeared to be the optimum frequency. The resistance of the solution was observed to increase owing to the death of microorganisms. First, the electrode was initially filled with buffer solution (with no bacteria) and subjected to impedance reading for 600 s to determine the baseline. Subsequently, solutions of bacteria (70 μL of bacteria solution is inserted into 7 mL of PBS) at 10^3^, 10^5^, and 10^7^ cfu/mL were incubated with the surface-coated electrode, and the impedance was measured for 2400 s. The impedance response was constantly scrutinized before and after injection of the *S. aureus* or *E. coli* samples. When we examined copolymer 2b we only observed 452 Ω increase at a concentration 10^7^ cfu/mLfor *E. coli,* while there was 291 Ω increase at the same concentration for *S. aureus* (Figure 5a and Figure 5b, respectively).

**Figure 6:**
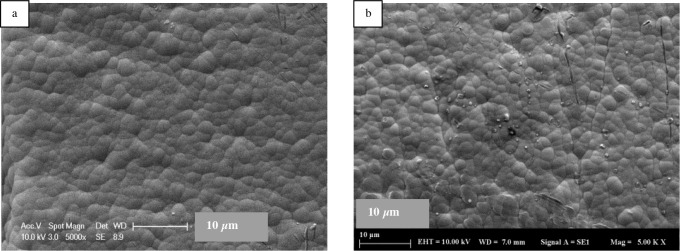
SEM images of (a) 3b coated electrode surface and (b) after deposition of S. aureus on the 3b coated electrode.

Impedance signal for *E. coli* was determinated for copolymer 3b at concentrations of 10^7^ cfu/mL (598 Ω increase) and 10^5^ cfu/mL (279 Ω increase) (Figure 5c). Highest impedance signals for *S. aureus* was observed for copolymer 3b at concentrations of 10^7^ cfu/mL (672 Ω increase) and 10^5^ cfu/mL (348 Ω increase), respectively (Figure 5d). We also observed that the signal is proportional to the concentration for both bacteria species tested. The difference in response may be caused by several factors, such as the threshold concentration for the adhesion and binding kinetics as a result of disruption of the bacterial membrane, which may be reflected in their respective electrical properties. We thought that the presence of the hexylpyridinium functionality enhanced the biocidal activity of the sensor surface and resulted in a better signal. After the impedance spectroscopic measurements, electrodes were conducted SEM analysis. Figure 6 shows the SEM images of deposition of *S. aureus* on the electrode surface. It was observed that because of the death of bacteria, organelles spread to the surface and the surface roughness increased, and small hillocks were formed.

The structural differences between *E. coli* and *S. aureus* make it likely that a difference will be observed in the impedance signals. It is well-known that the membrane compositions of Gram-negative bacteria ( *E. coli* ) and Gram-positive bacteria ( *S. aureus* ) are different. The cytoplasm of *E. coli* is surrounded by a phospholipid inner membrane, a peptidoglycan wall, and an anionic outer lipid membrane. In contrast, the cytoplasm of *S. aureus* is surrounded by a lipid membrane and a peptidoglycan layer. Therefore, the electrical properties of *S. aureus* and *E. coli* with their different membrane and cytoplasm structures are different owing to their different conducting characteristics [10,53]. Gram-positive bacteria generally have a higher K^+^ content than Gram-negative bacteria, which explains the higher internal conductivity of *S. aureus* than *E. coli* [53]. Although electrolytic effects become significant at low frequencies (<1 kHz), adhesion kinetics on the polymer sensor and the occurrence of anionic phospholipids from dead bacteria might also play a crucial role in the impedance signal. 

The different impedimetric responses for *E. coli* and *S. aureus* can also be attributed to the different binding receptors on their surfaces. For example, man-PTSs receptors in both Gram-positive and Gram-negative bacteria bind to antibacterial peptides and bacteriophages [54,55]. Deviations in the local concentrations and expression levels of the receptors result in different binding sensitivities on the bactericidal electrode surface and lead to changes in impedance sensor responses [11]. Adhered bacterial cells seem to interact nonspecifically with the polymer sensor and result in strong impedance changes, giving rise to an increase in the resistance. The sensitivity of polymer coated sensor depends on several factors such as the buffer concentration or media temperature, or bacteria concentration. In order to better control the conductivity, the experiment could be performed with diluted PBS solution, but more work is needed to optimize the conditions and investigate the reasons for the differences in the impedance signals. 

### 3.7. Cyclic voltammetry

The cyclic voltammetry (CV) method is a useful method in examining the electron transfer property between the sensor surface and the species in solution [56,57]. In this study, Fe(CN)_6_^3–/4– ^redox pair was chosen as the probe in order to examine the bacterial adsorption on the polymer film coated on the electrode surface. As the penetration of the redox probe to the electrode surface and the charge transfer efficiency of the electrode decrease with the bacterial adsorption, a decrease in the reduction/oxidation peak intensity of the Fe(CN)_6_^3–/4– ^redox pair and an increase between the peak potentials are expected. In the CV study, IDEA, Pt wire, and calomel (3 M KCl) electrode were used as the working, auxiliary and reference electrodes, respectively. 0.01 M K_4_[Fe(CN)_6_] solution was prepared in PBS. Cyclic voltammetry in the range of –400–(+ 800) mV at a scanning speed of 100 mV s^–1^ were recorded with IDEA electrodes coated with 3b polymer. After that, the solution of *S. aureus* and *E.coli* with two different concentrations 10^5^ and 10^7^ cfu/mL, respectively, was added into the CVs experiment set up under the same condition and recorded for 15 min. 

**Figure 7:**
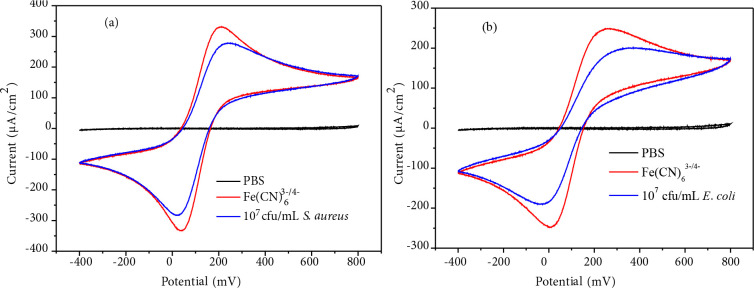
(a) CV of 3b-coated electrode in the presence of 107 cfu/mL S. aureus (b) CV of 3b-coated electrode in the presence of 10^7^ cfu/mL E. coli.

Figure 7 shows that the peak intensity decreases and the difference between peak potentials increases after bacteria solutions are inserted. The intensity of the oxidation and reduction peaks at approximately 200 mV and 60 mV in the 3b coated electrode decreases with *E.coli* adsorption. The same trend is also observed for *S. aureus,* with slightly lower response than the *E.coli* . We thought that *E. coli* adsorbed on the surface of polymer coated electrodes more efficiently and could form an insulating layer to diminish the current. 

CVs of the polymer coated electrode against 10^5^ cfu/mL bacteria solution were also performed. (Figure S28 and S29) However slight change in the current was observed after the insertion of bacteria solution. This result parallel with the impedance spectrum which was discussed above, and we conclude that the detection limit of bacteria is 10^7^cfu/mL. Thresholds concentration of bacteria is important that might prevent the penetration of the redox probe to the electrode and thus result in a decrease in the charge transfer efficiency.

### 3.8. Real-time detection in milk sample

For proving the applicability of the polymer 3b coated sensor array in real-life applications, we have used it for detection of bacteria in contaminated milk samples. For this purpose, 70 μL of *S. aureus* or *E. coli* solution was inserted into 7 mL of milk sample, and then electrode was inserted into these solutions. In a separate experiment, we incubated electrode first in milk solution for 600 s; after that time then 70 μL of *S. aureus* solution was inserted into this solution. Impedance-time graph of the 3b coated electrode against the bacteria contaminated milk sample showed that electrode did not give a high impedance response (Figure 30). When we compare milk with PBS, milk has a very high molecular density due to the fats, sugars, enzymes, and proteins it contains. These components, unfortunately, can attach to the electrode surface before bacteria can and lead to false positive or negative results. The same result has been previously shown that proteins, enzymes, fats, components of milk affect impedance response of the interdigitated electrodes and conduce towards an increase in resistance [11,48–50]. However, serial dilution with PBS could enhance the proper impedance signal.

## 4. Conclusion

In this work, we presented a new method for the detection of *S. aureus* and *E. coli* using an impedimetric biosensor platform developed based on real-time, label-free detection using antimicrobial cationic polymers derived from ROMP. In the first part of the study, different cationic block and random copolymers were synthesized, and their biocidal activities were analyzed on a glass surface. This active polymer was then used to coat a gold electrode, which was applied for the detection of bacteria in buffer. The biosensor was able to detect Gram-positive and Gram-negative bacteria with a limit of detection (LOD) of 10^7^ cfu/mL. However, the signal to noise ratio needs to be increased to achieve a better LOD with sufficient sensitivity.

These results suggest that the polymer-coated electrode needs to be optimized to improve its sensitivity. Higher molecular weight polymers with different cationic or carbohydrate functionalities and hydrophobicities could enhance electrode performance. We will carry out further investigations to improve the electrode performance for bacterial detection. 

Supplementary Materials
