# Supplementary material for: Detection of bacteria using antimicrobial polymer derived via ring-opening metathesis (romp) pathway
Source: Turk J Chem. 2021 Aug 27;45(4):986–1003. doi: 10.3906/kim-2012-14 (PMC8517495; doi:10.3906/kim-2012-14)
Supplement: Supplementary file 1 — Supplementary Materials [file turkjchem-45-986-sup001.pdf]

**Supporting information**

## 1. Monomer and polymer synthesis

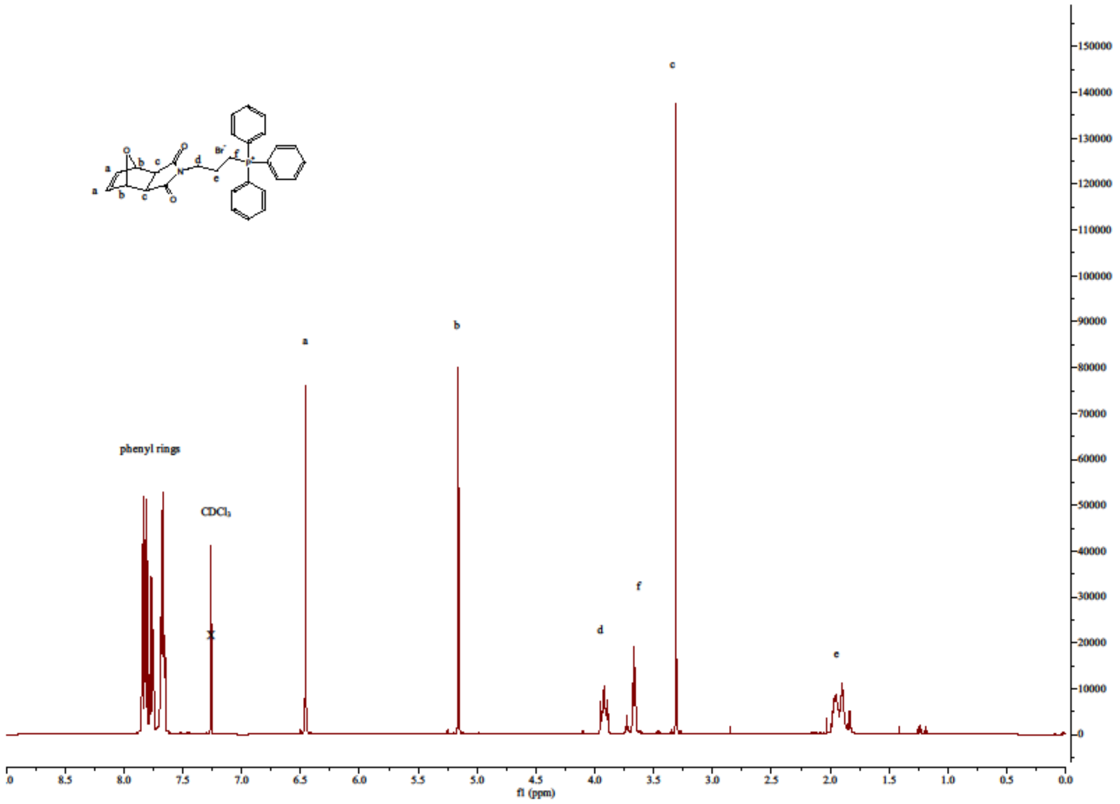

**Figure S1.** $^1H$ NMR spectrum of monomer 1 in $CDCl_3$.

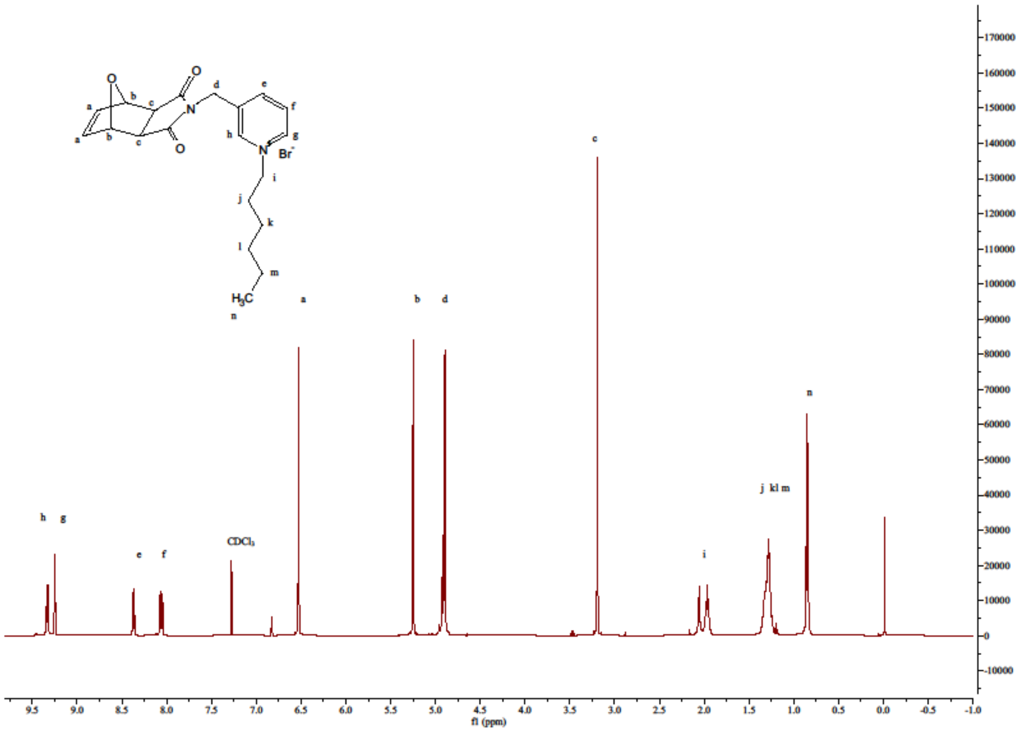

**Figure S2.** $^{1}$H NMR spectrum of monomer 2 in $CDCl_3$.

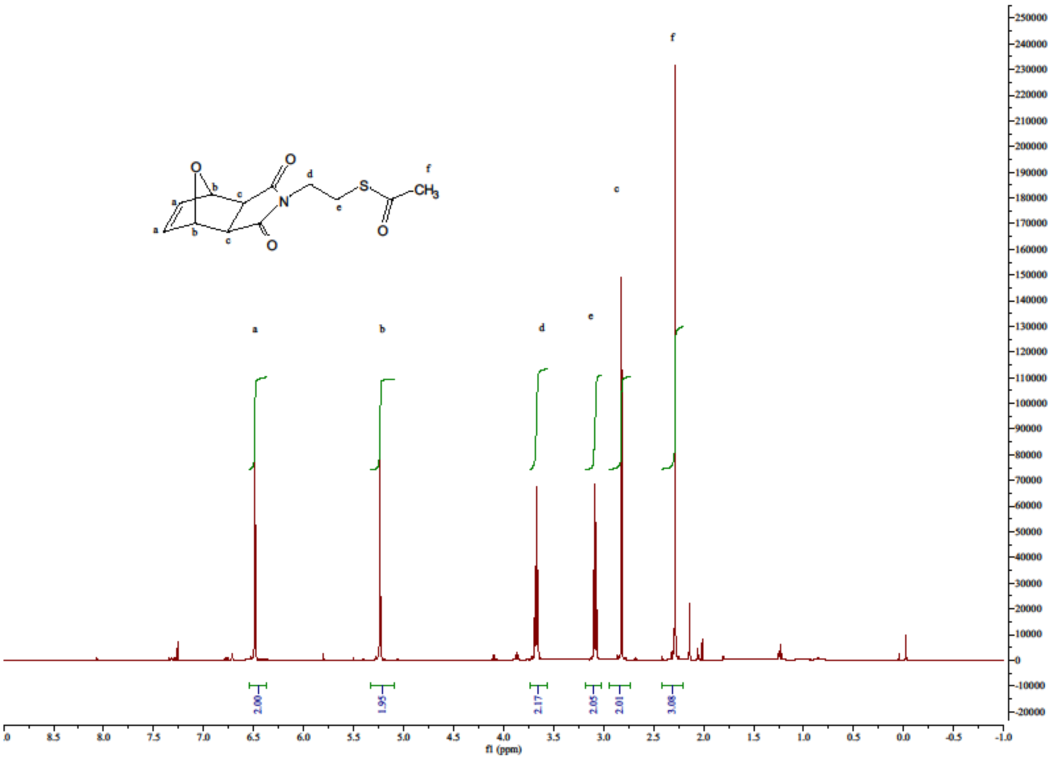

**Figure S3.** $^{1}$H NMR spectrum of monomer 3 in $CDCl_3$.

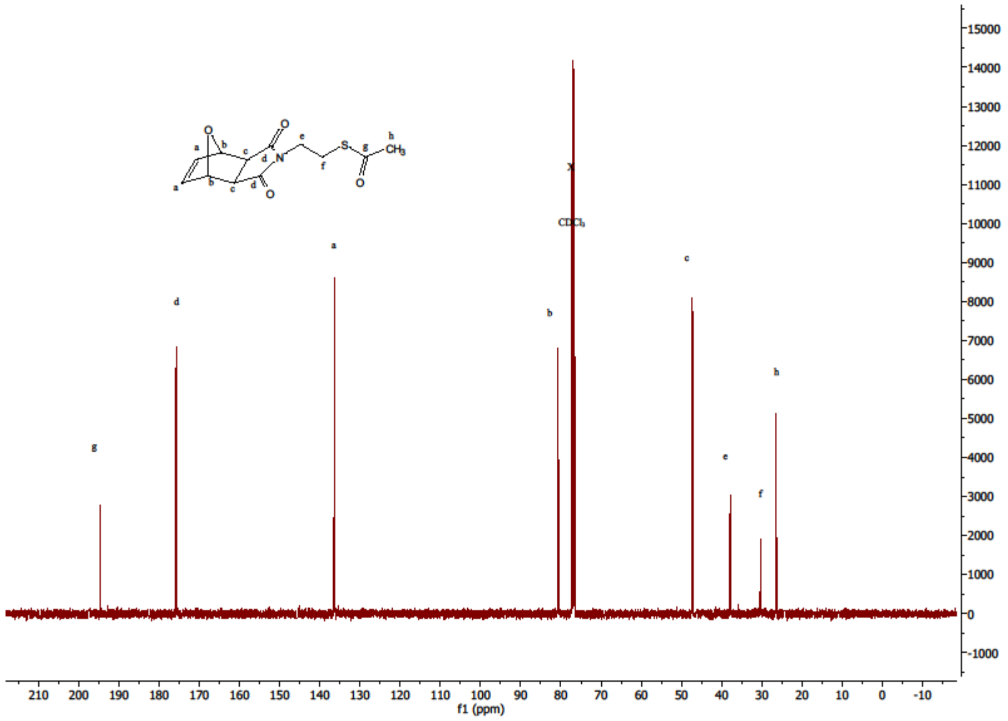


**Figure S4.** $^{13}$C NMR spectrum of monomer 3 in $CDCl_3$.

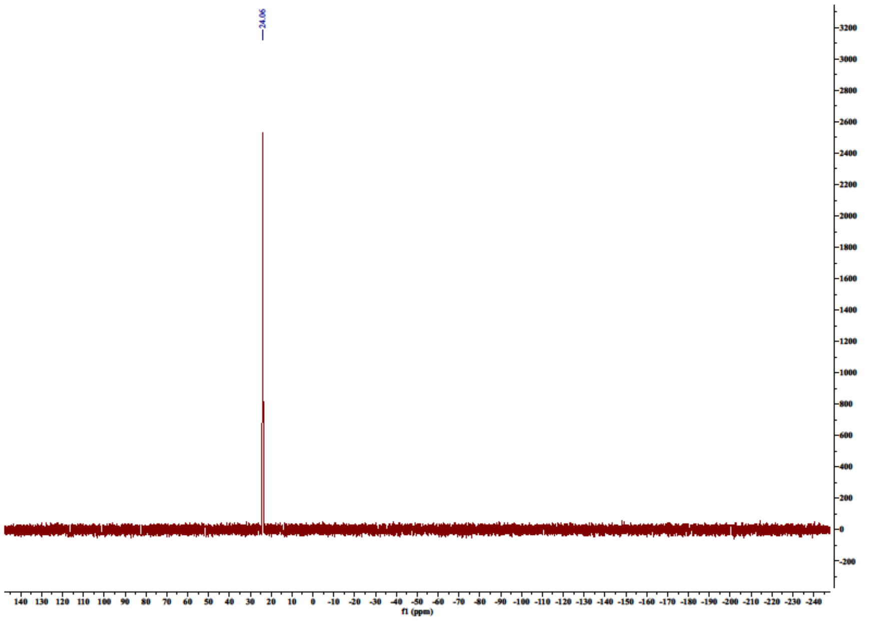


**Figure S5.** $^{31}$P NMR spectrum of block copolymer 2a ($M_{n,th}$ = 10 000 g/mol with a theoretical ratio m:n (8:2) by weight) in DMSO-$d_6$.

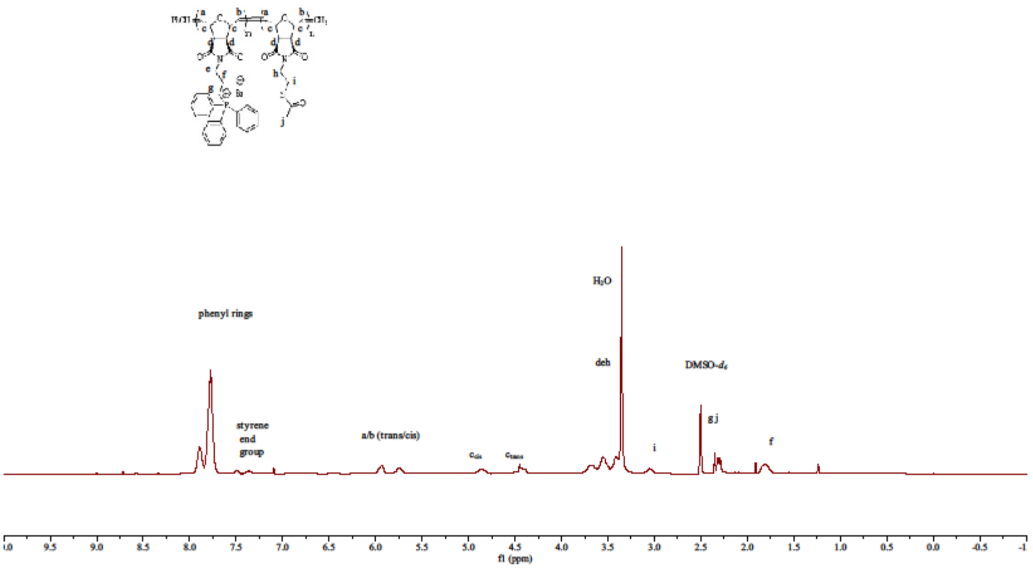


**Figure S6.** $^{1}$H NMR spectrum of block copolymer 2b ($M_{n,th}$ = 3 000 g/mol; with a theoretical ratio m:n (8:2) by weight) in DMSO-$d_6$.

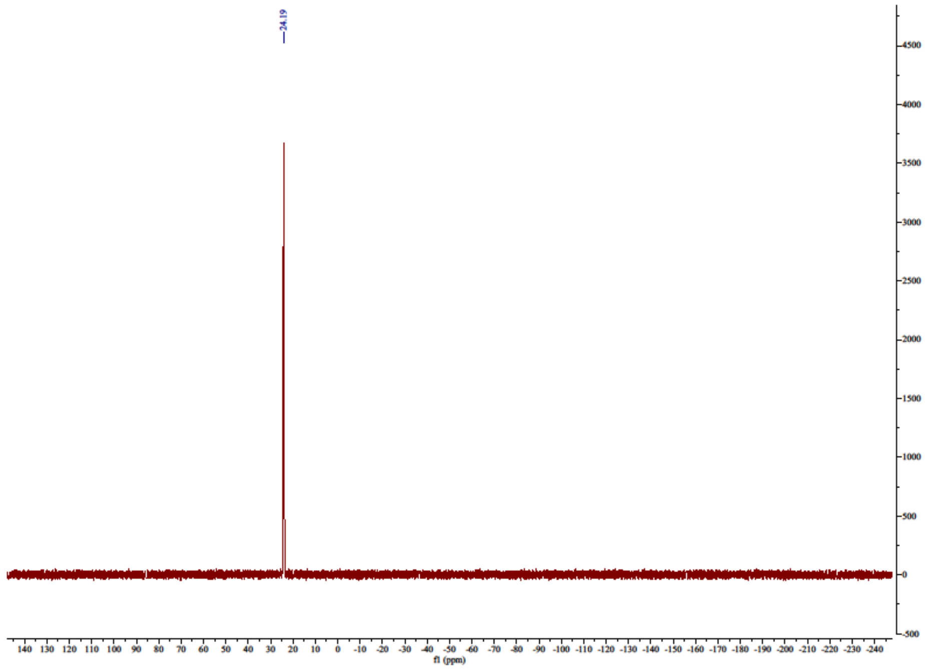


**Figure S7.** $^{31}$P NMR spectrum of block copolymer 2b ($M_{n,th}$ = 3000 g/mol; with a theoretical ratio m:n (8:2) by weight) in DMSO-d$_6$.

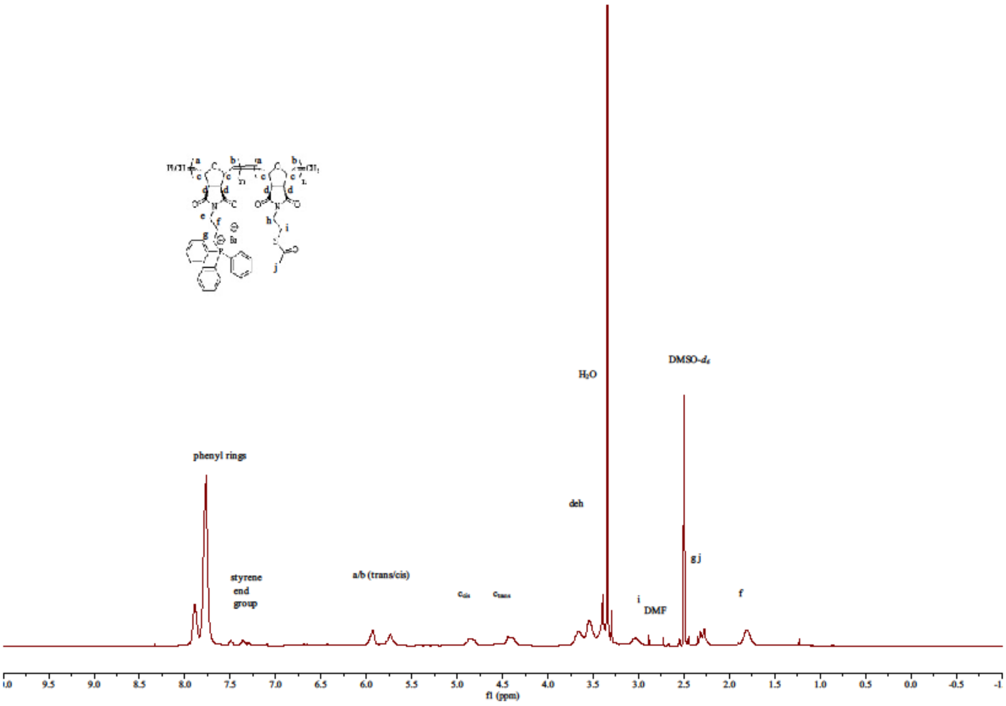


**Figure S8.** $^{1}$H NMR spectrum of random copolymer 2c ($M_{n,th}$ = 3000 g/mol; with a theoretical ratio m:n (8:2) by weight) in DMSO-$d_6$.

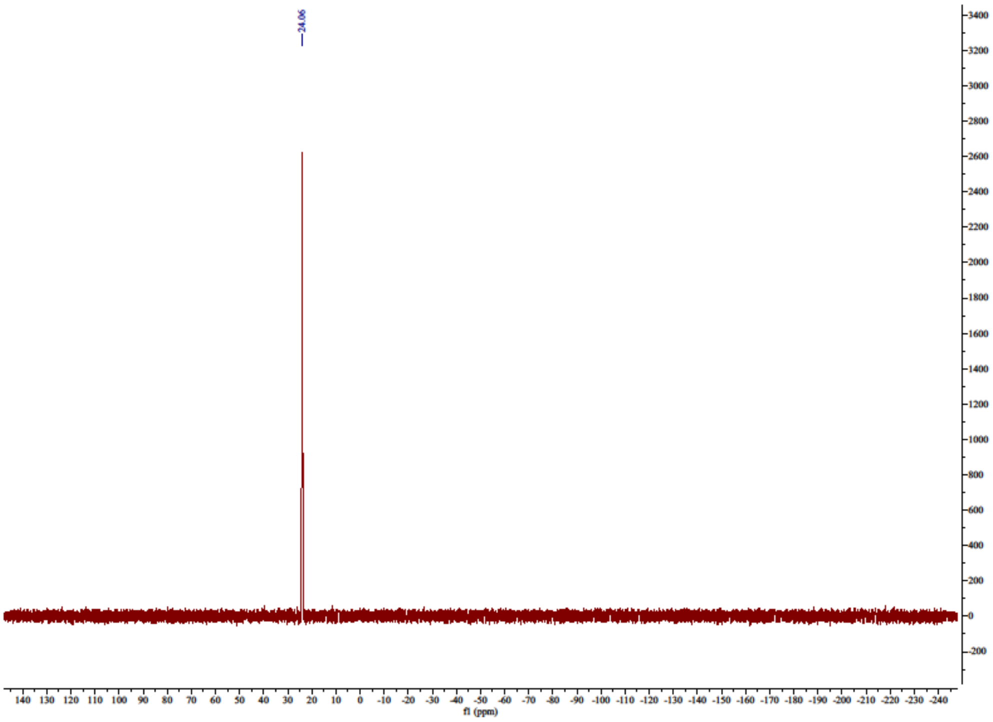


**Figure S9.** $^{31}$P NMR spectrum of random copolymer 2c ($M_{n,th}$ = 3000 g/mol; with a theoretical ratio m:n (8:2) by weight) in DMSO-$d_6$.

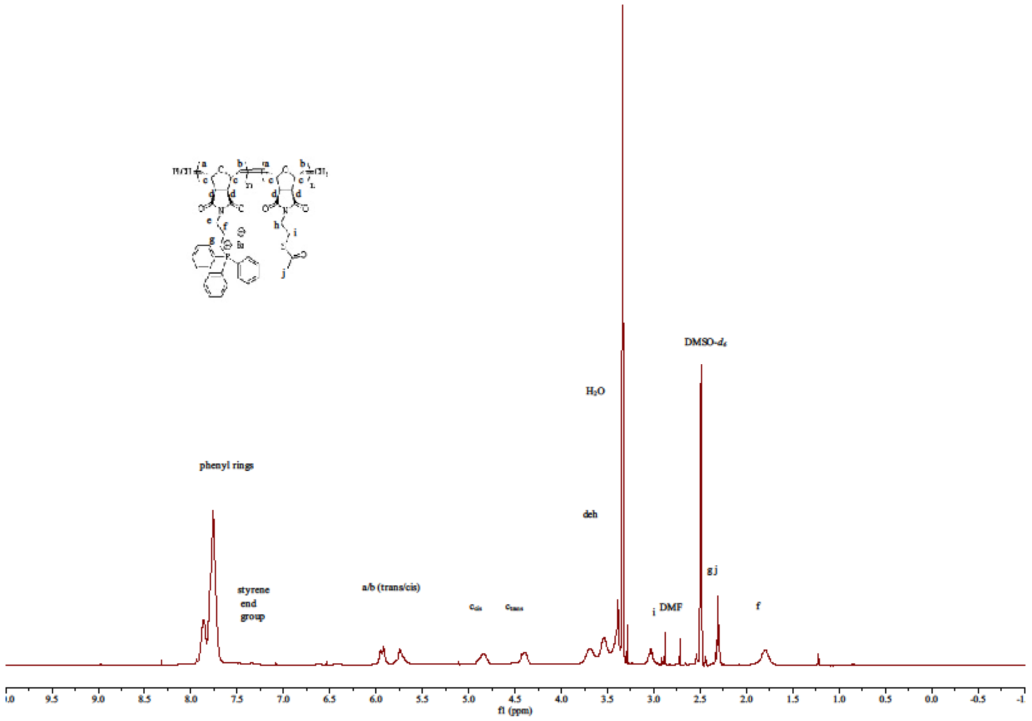


**Figure S10.** $^{1}$H NMR spectrum of block copolymer 2d ($M_{n,th}$ = 10,000 g/mol; with a theoretical ratio m:n (7:3) by weight) in DMSO-$d_6$.

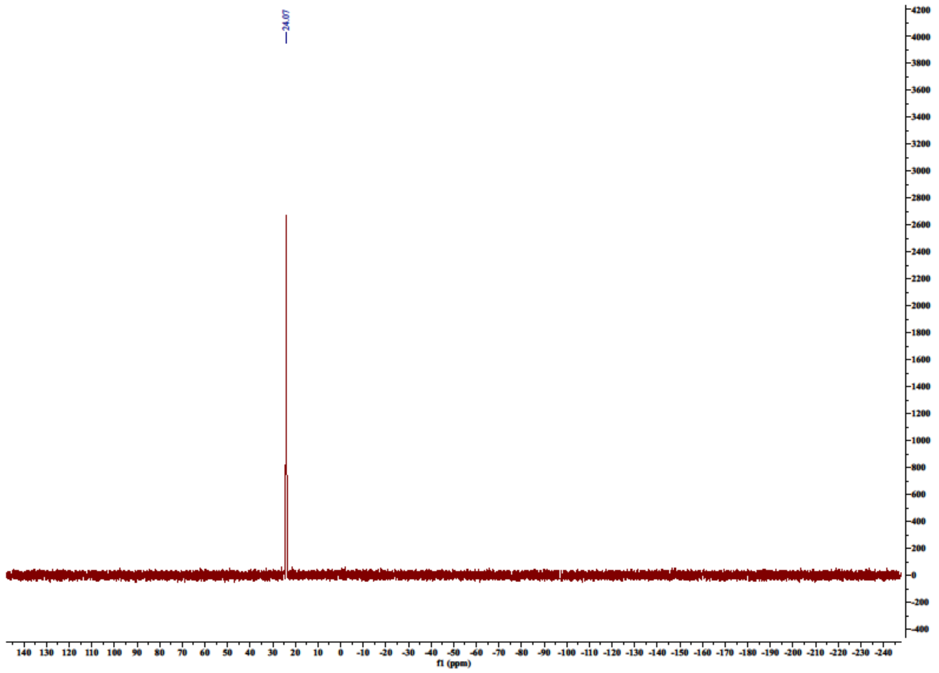


**Figure S11.** $^{31}$P NMR spectrum of block copolymer 2d ($M_{n,th}$ = 10,000 g/mol; with a theoretical ratio m:n (7:3) by weight) in DMSO-$d_6$.

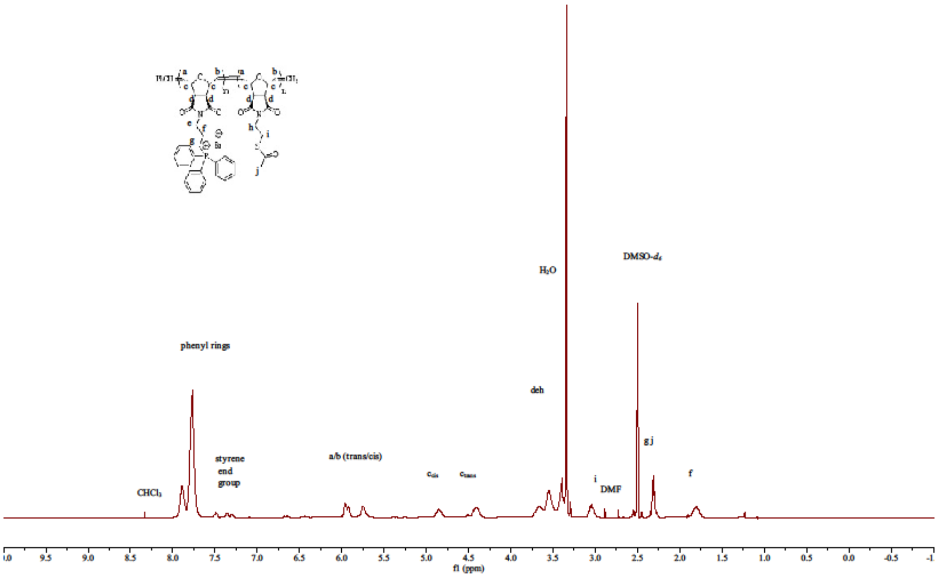


**Figure S12.** $^{1}$H NMR spectrum of block copolymer 2e ($M_{n,th}$ = 3000 g/mol; with a theoretical ratio m:n (7:3) by weight) in DMSO-*d*$_6$.

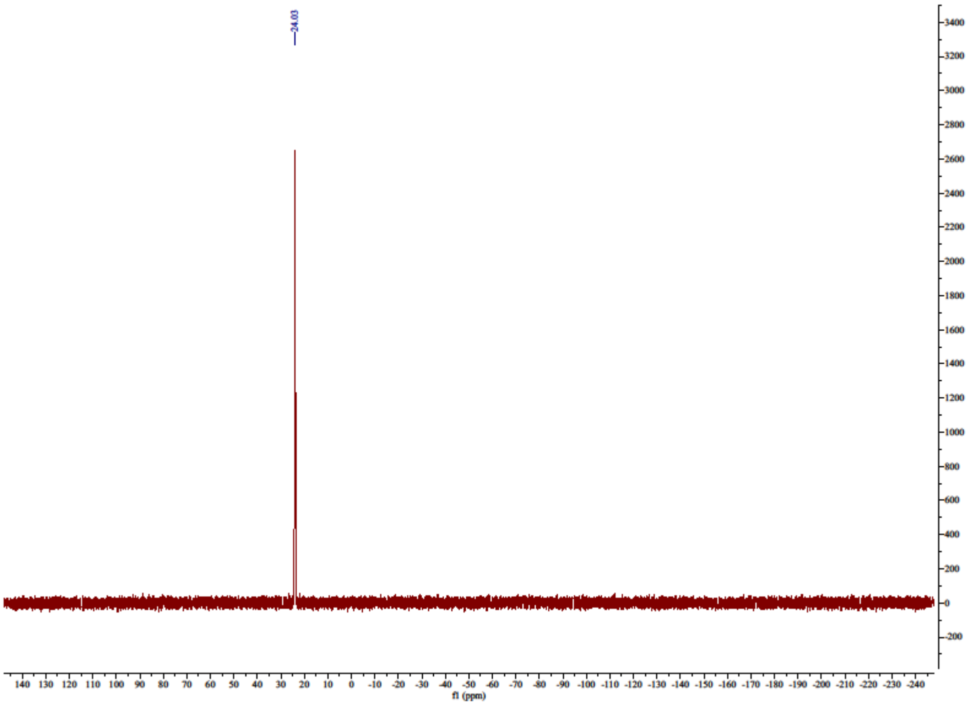


**Figure S13.** $^{31}$P NMR spectrum of block copolymer 2e ($M_{n,th}$ = 3000 g/mol; with a theoretical ratio m:n (7:3) by weight) in DMSO-*d*$_6$.

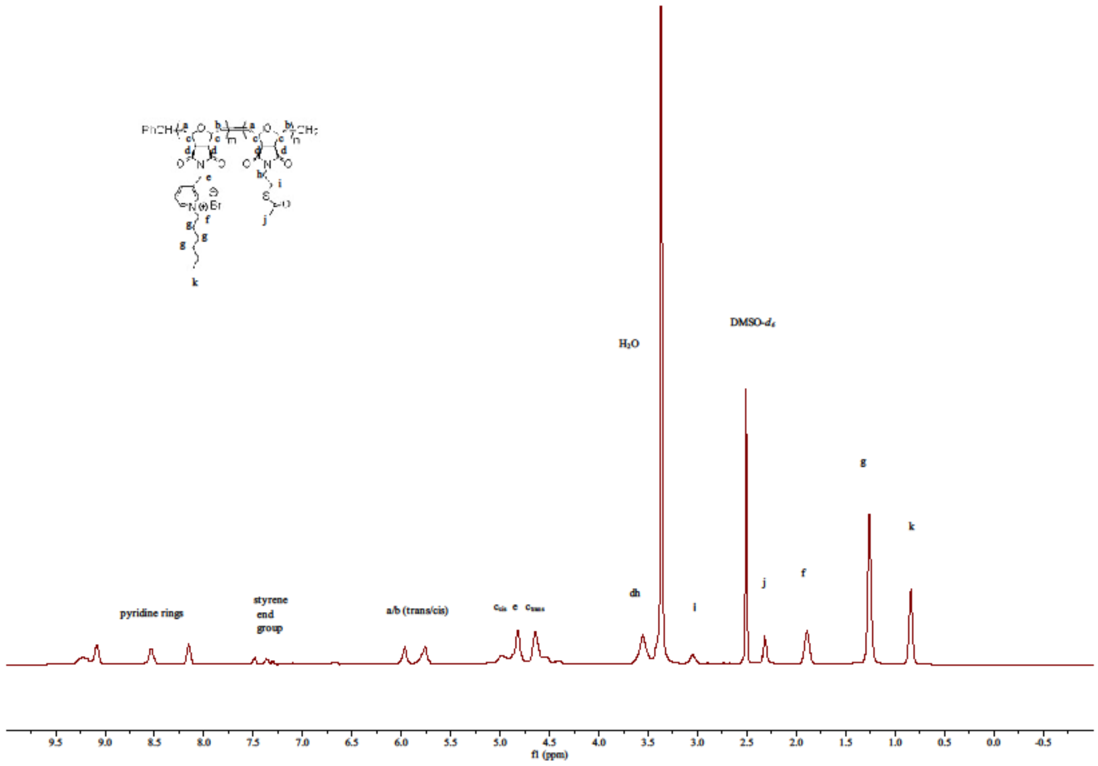


**Figure S14.** $^{1}$H NMR spectrum of block copolymer 3b ($M_{n,th}$ = 3000 g/mol; with a theoretical ratio m:n (8:2) by weight) in DMSO-$d_6$.

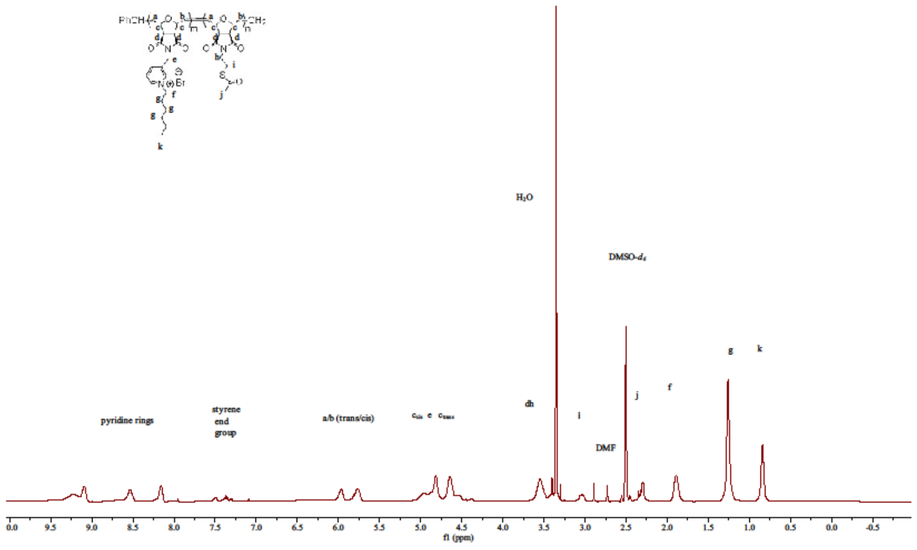


**Figure S15.** $^{1}$H NMR spectrum of random copolymer 3c ($M_{n,th}$ = 3000 g/mol; with a theoretical ratio m:n (8:2) by weight) in DMSO-$d_6$.

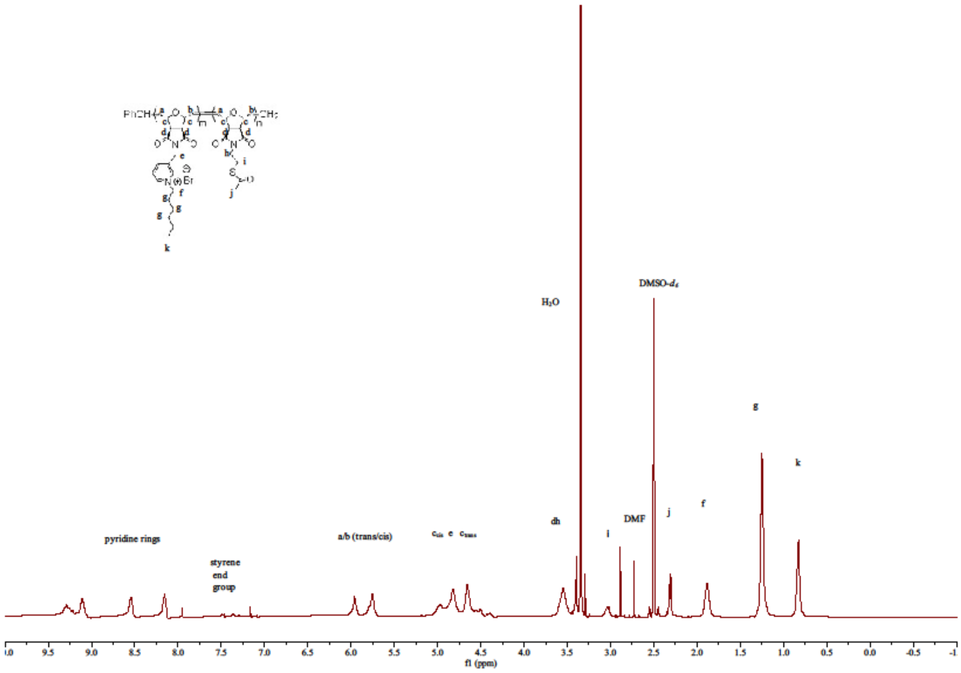


**Figure S16.** $^{1}$H NMR spectrum of block copolymer 3d ($M_{n,th}$ = 10,000 g/mol; with a theoretical ratio m:n (7:3) by weight) in DMSO-$d_6$.

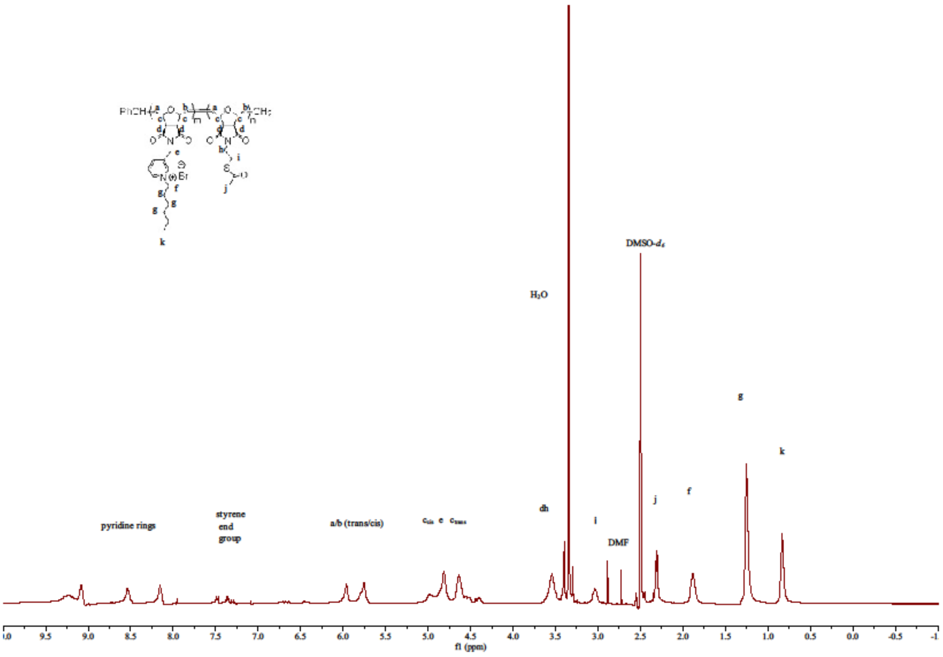


**Figure S17.** $^{1}$H NMR spectrum of block copolymer 3e ($M_{n,th}$ = 3000 g/mol; with a theoretical ratio m:n (7:3) by weight) in DMSO-$d_6$.

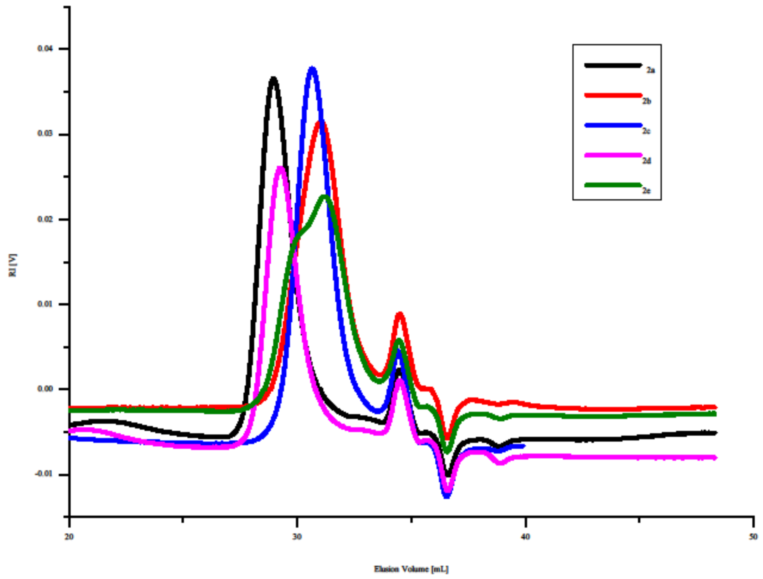


**Figure S18.** GPC graph of polymers (2a-e).

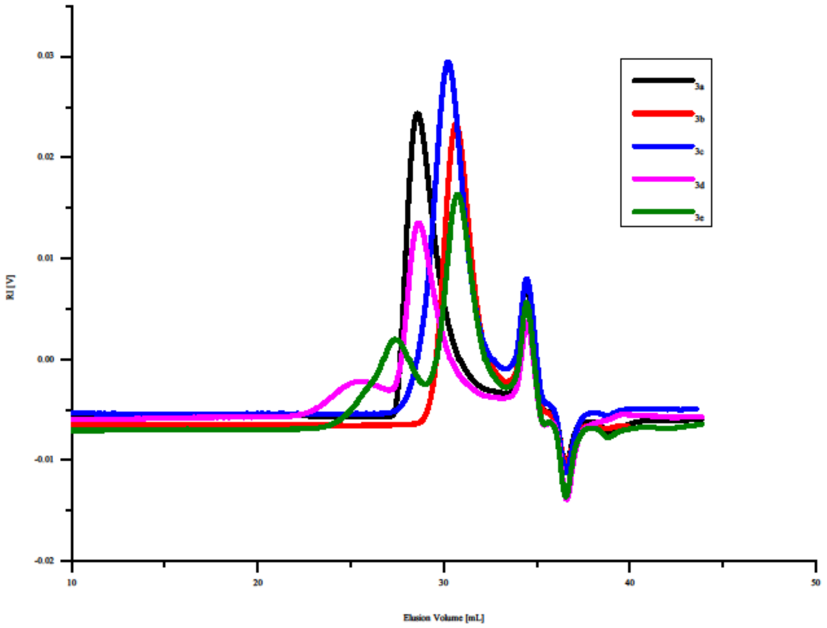


**Figure S19.** GPC graph of polymers (3a-e).

## 2. Determination of the solid phase antibacterial activity

The results are determined as;

log reduction = log (cell count of control) – log (survivor count on test samples glass materials).

$$\text{kill \%} = \frac{\text{cell count of control} - \text{survivor count on test samples glass materials}}{\text{cell count of control}} \text{ x } 100.$$

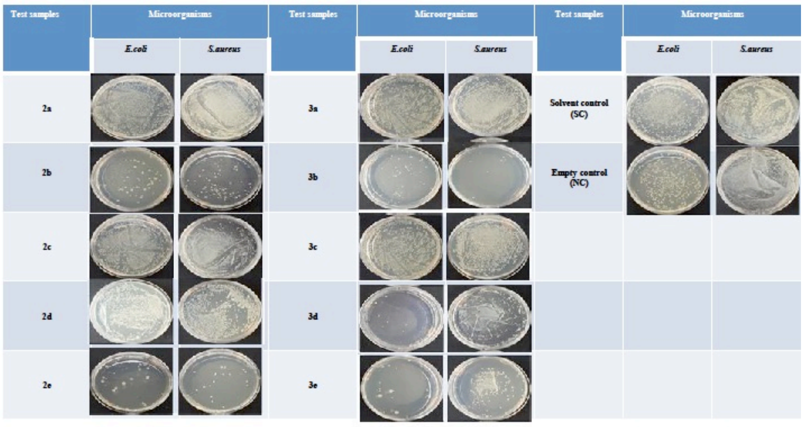


**Figure S20**. The petri images of surviving bacteria after exposure with test samples. (ATCC is the registered trademark of American Type Culture Collection).

## 3. Impedance spectroscopy analysis

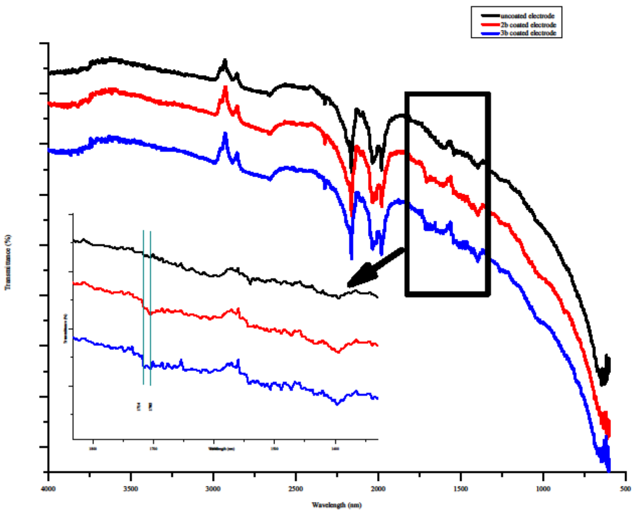


**Figure S21.** FTIR spectrum of uncoated (bare), 2b and 3b coated electrode.

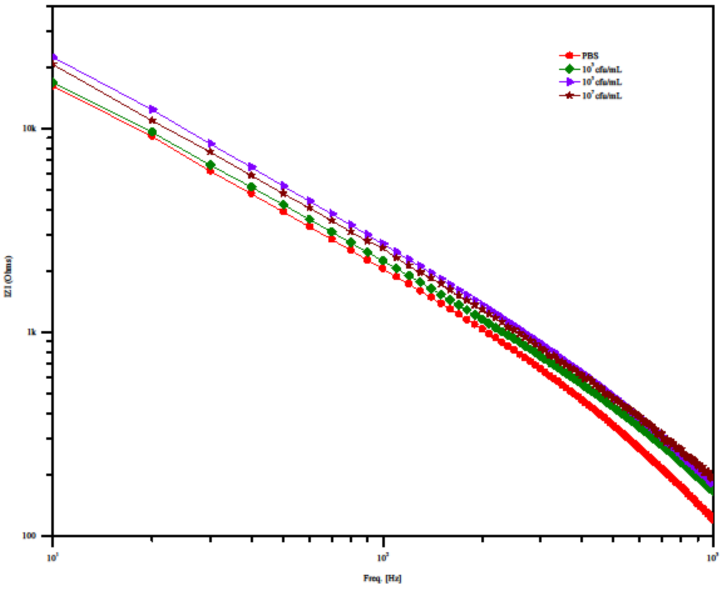


**Figure S22.** Impedance-frequency graph of the polymer 2b coated electrode against various concentrations of *E. coli*.

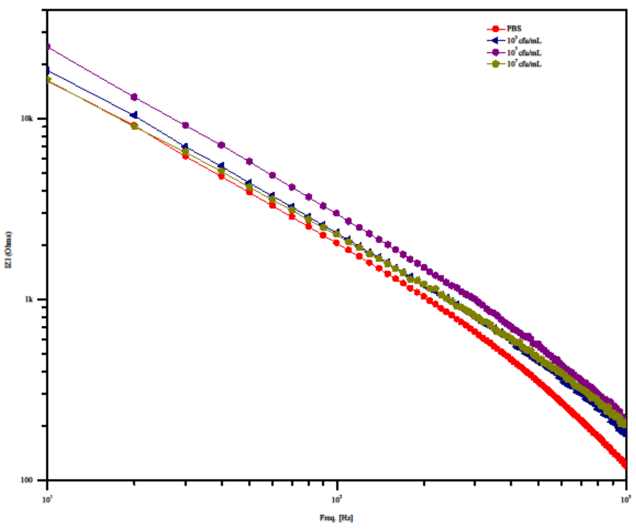


**Figure S23.** Impedance-frequency graph of the polymer 2b coated electrode against various concentrations of *S. aureus*.

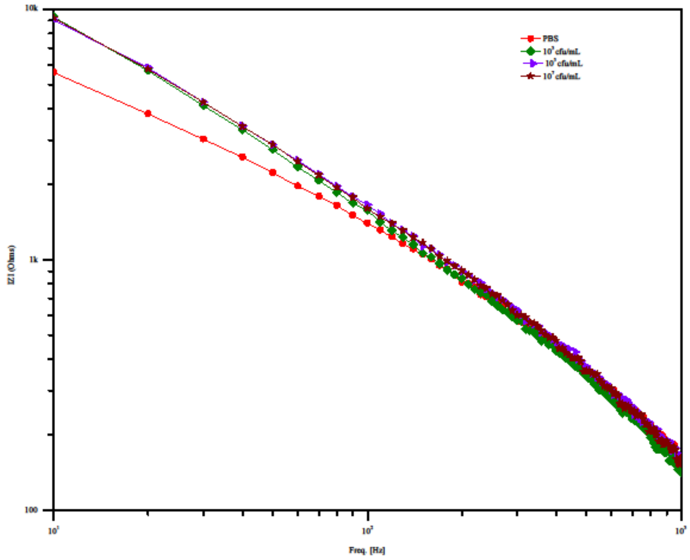


**Figure S24.** Impedance-frequency graph of the polymer 3b coated electrode against *E.coli* with different concentration.

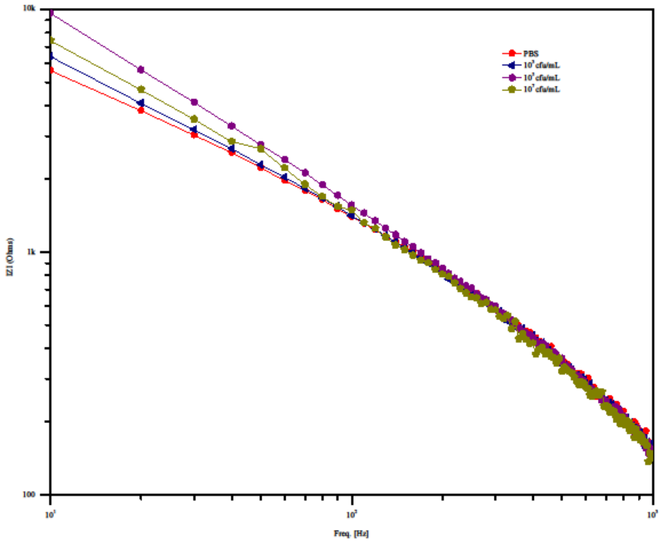


**Figure S25.** Impedance-frequency graph of the polymer 3b coated electrode against *S. aureus* with different concentration.

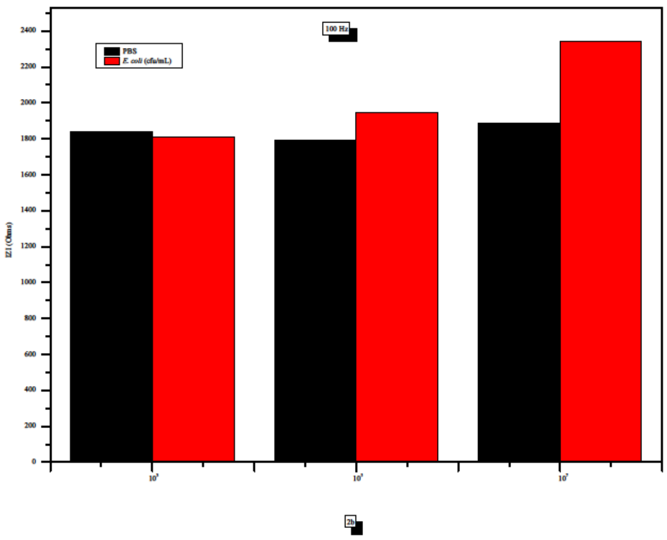


(a)

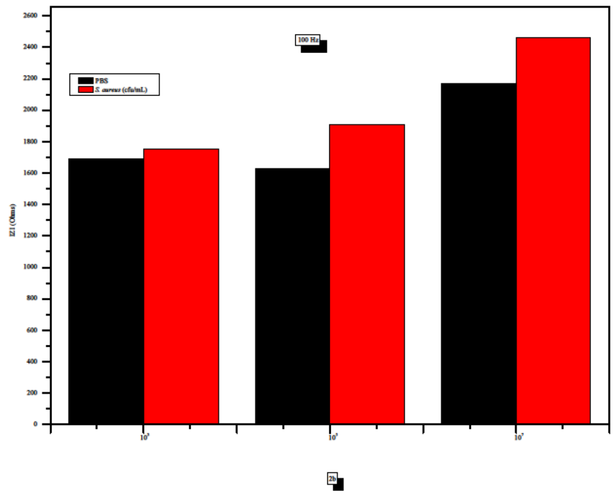


(b)

**Figure S26.** The real-time impedimetric response of the antibacterial polymer sensor 2b to various concentrations of (a) *E. coli* and (b) *S. aureus* at a fixed frequency (100 Hz).

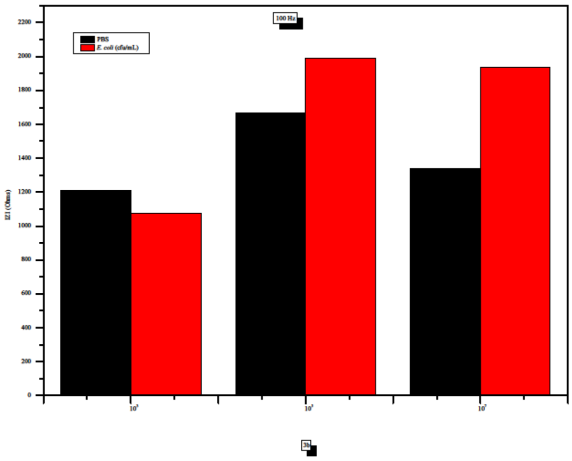


(a)

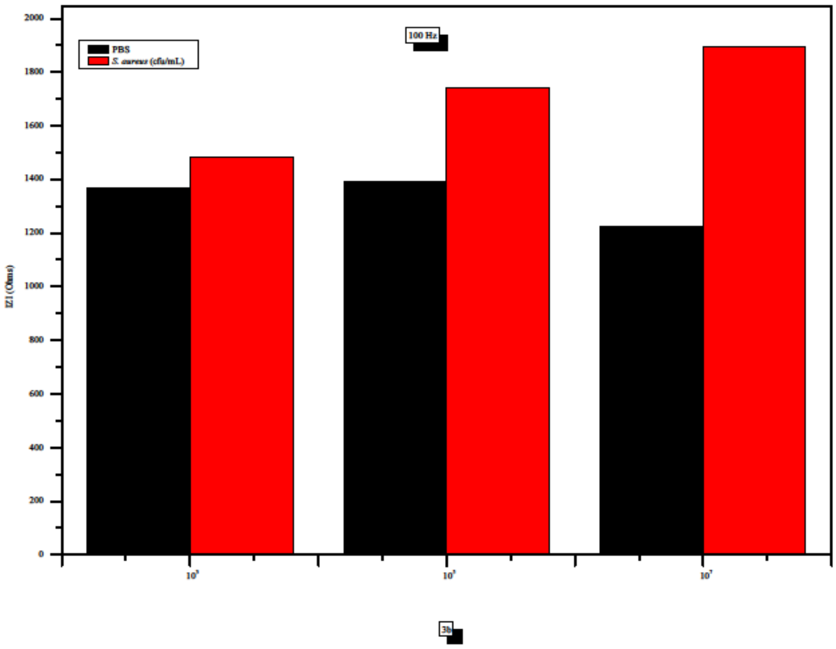


(b)

**Figure S27.** The real-time impedimetric response of the antibacterial polymer 3b sensor response to various concentrations of (a) *E. coli* and (b) *S. aureus* at a fixed frequency (100 Hz).

## 4. Cyclic voltammetry

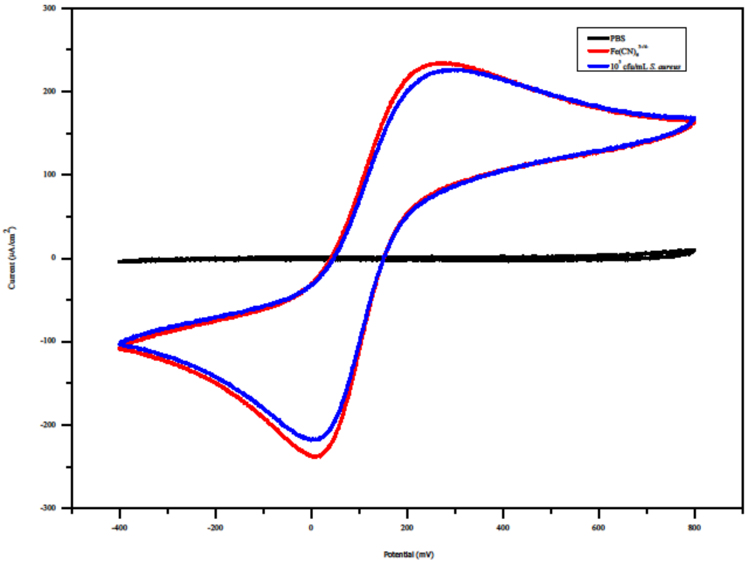


**Figure S28.** CV of polymer 3b in the presence of $10^5$ cfu/mL *S.aureus*.. (━): PBS (━):0.01 M $Fe(CN)_6^{3-/4-}$ (━): after exposed to $10^5$ cfu/mL *S. aureus*.

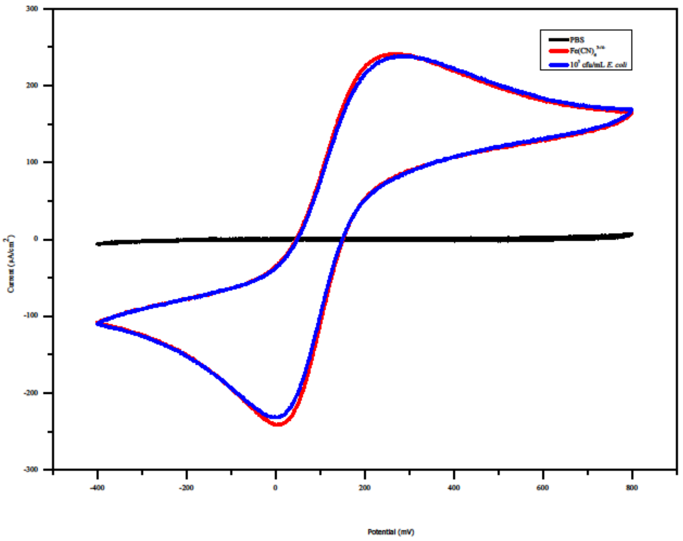


**Figure S29.** CV of polymer 3b in the presence of $10^5$ cfu/mL *E.coli*. (▬): PBS, 0.01 M $Fe(CN)_6^{3-/4-}$ redox probe (▬): before and (▬): after exposed to $10^5$ cfu/mL *E coli*.

## 5. Real-time detection in milk sample

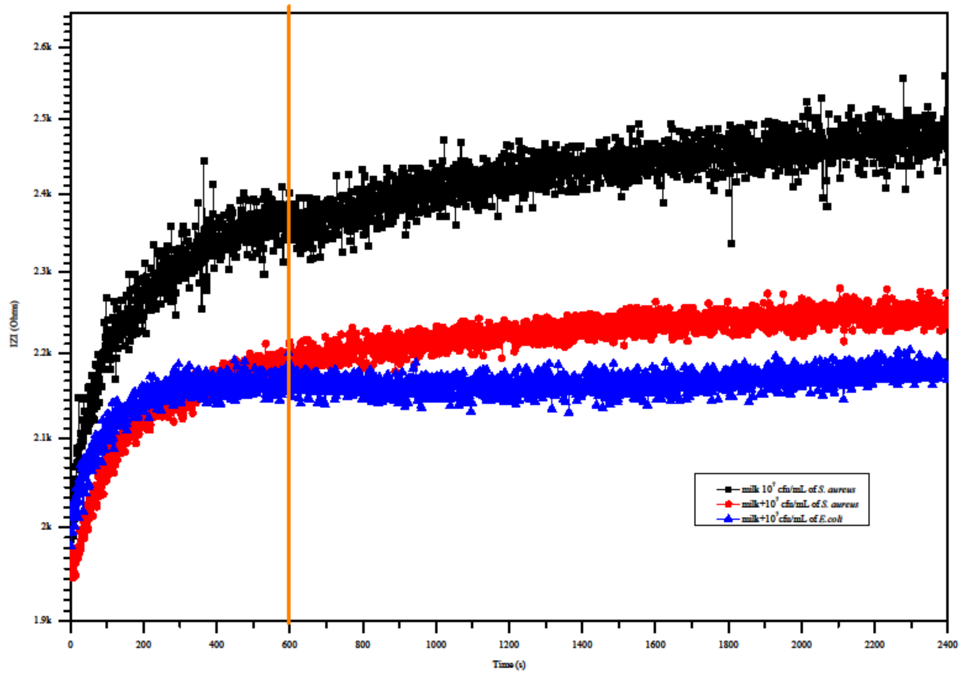


**Figure S30.** Impedance-time graph of the polymer 3b coated electrode against the bacteria contaminated milk sample (▬): *S. aureus* was added 600 s later at a concentration of $10^7$ cfu/mL (▬): *S. aureus* contaminated milk sample was measured (▬):*E. coli* contaminated milk sample was measured.
